# Quality of life over time after new onset refractory status epilepticus

**DOI:** 10.1111/epi.18635

**Published:** 2025-09-13

**Authors:** Matthew D. Gruen, Margaret T. Gopaul, Anthony D. Jimenez, Ayush Batra, Leah J. Blank, Charlotte Damien, Gregory S. Day, Krista Eschbach, Elizabeth E. Gerard, Teneille E. Gofton, Stephen T. Hantus, Nathalie Jette, Amy Jongeling, Peter Kang, Karnig Kazazian, Marissa Kellogg, Minjee Kim, Bahar Madani, Mikaela Morales, Vineet Punia, Claude Steriade, Aaron Struck, Olga Taraschenko, Nathan Torcida, Mark S. Wainwright, Ji Yeoun Yoo, Nicolas Gaspard, Nora Wong, Lawrence J. Hirsch, Aurélie Hanin

**Affiliations:** ^1^ Department of Neurology, Comprehensive Epilepsy Center Yale University School of Medicine New Haven Connecticut USA; ^2^ Feinberg School of Medicine Northwestern University Chicago Illinois USA; ^3^ Icahn School of Medicine at Mount Sinai New York New York USA; ^4^ Department of Neurology, Hôpital Universitaire de Bruxelles–Hôpital Erasme Université Libre de Bruxelles Brussels Belgium; ^5^ Department of Neurology Washington University School of Medicine Saint Louis Missouri USA; ^6^ Department of Neurology Mayo Clinic in Florida Jacksonville Florida USA; ^7^ Section of Neurology, Department of Pediatrics Children's Hospital Colorado, University of Colorado School of Medicine Aurora Colorado USA; ^8^ London Health Sciences Center, University Hospital London Ontario Canada; ^9^ Epilepsy Center Neurological Institute, Cleveland Clinic Cleveland Ohio USA; ^10^ Department of Clinical Neurosciences University of Calgary Calgary Alberta Canada; ^11^ Department of Neurology NYU Langone Medical Center New York New York USA; ^12^ Oregon Health & Science University Portland Oregon USA; ^13^ Division of Pediatric Neurology University of Washington Seattle Washington USA; ^14^ Department of Neurology University of Wisconsin Madison Wisconsin USA; ^15^ Department of Neurological Sciences University of Nebraska Medical Center Omaha Nebraska USA; ^16^ NORSE Institute Summit New Jersey USA; ^17^ Sorbonne Université, Institut du Cerveau ‐ Paris Brain Institute ‐ ICM, Inserm, CNRS, AP‐HP, Hôpital de la Pitié Salpêtrière Paris France; ^18^ Department of Metabolic Biochemistry, DMU BioGeMH, AP‐HP, Sorbonne Université, Pitié‐Salpêtrière‐ Charles Foix University Hospital Paris France

**Keywords:** new onset refractory status epilepticus, outcome, quality of life

## Abstract

**Objective:**

This study aims to better characterize the long‐term neurological quality of life (QOL) outcomes (using the Neuro‐QOL scale) in survivors of new onset refractory status epilepticus (NORSE), including its subtype febrile infection‐related epilepsy syndrome (FIRES), and provide guidance for psychological and social support strategies.

**Methods:**

Utilizing data from a multicenter prospective study of NORSE/FIRES led by Yale University, we enrolled patients who completed the validated, patient‐reported Neuro‐QOL scale at least once at 3–6 months (*n* = 37), 12 months (*n* = 29), 24 months (*n* = 23), or ≥36 months (*n* = 9) following discharge. The Neuro‐QOL scale assesses physical, mental, and social health in patients with neurological disorders. QOL impairment (QOL‐I) scores were calculated, with higher scores indicating greater impairment. *T*‐scores enabled comparisons with reference populations.

**Results:**

In adults, median QOL‐I improved from 44.1% at 3–6 months to 37.6% at 36+ months. Paired analysis showed significant improvement in QOL‐I between 3–6 and 24 months (*p* = .016), with specific improvements in communication, satisfaction with social roles, fatigue, and mobility. Greater improvement was also observed for participation in social roles (5.5‐point *T*‐score gain) compared to the reference population, suggesting meaningful change. A gradual improvement in overall QOL‐I scores was also observed in pediatric participants, despite a modest sample size (*n* = 5 with data at 3–6 and 12 months). Measures of fatigue and anxiety persisted in adults, and cognitive difficulties persisted in both adults and children. In adults, longer status epilepticus duration and intensive care unit stay were associated with poorer QOL. Additionally, a higher number of antiseizure medications was associated with more depression, cognitive impairments, and perceived stigma.

**Significance:**

These findings highlight the potential for recovery following an acute episode of NORSE, although many patients continue to face challenges requiring ongoing support, and the clinical meaning of the reported QOL improvement remains unclear. Furthermore, the findings underscore the importance of strategic multidisciplinary support systems in the years following discharge.

**Key points**

- In adults, fatigue was common early on, whereas anxiety and cognitive issues increased by 24 months.
- Adult quality of life measures improved over time, especially for fatigue, social roles, and communication.
- Children's quality of life measures improved, with varied emotional and cognitive challenges.
- Longer status epilepticus duration and ICU stay were associated with worse quality of life, including more fatigue and cognitive problems.
- The first 12 months are likely the most critically important for recovery.

## 1 INTRODUCTION

New onset refractory status epilepticus (NORSE) is one of the most severe forms of refractory status epilepticus (RSE), associated with high mortality and morbidity. NORSE occurs in children or adults with no prior history of epilepsy or relevant neurological disorders and in whom no clear acute or active metabolic, toxic, or structural cause is identified within the first few days.^1^ Febrile infection‐related epilepsy syndrome (FIRES) is a subset of NORSE that is characterized by the occurrence of a febrile illness between 2 weeks and 24 h before status epilepticus (SE) onset.^1^ Although extensive workups may reveal an underlying etiology (e.g., autoimmune encephalitis, viral encephalitis, genetic disorders), more than 50% of cases remain of unknown origin (even higher in children) and are classified as cryptogenic.^2^, ^3^, ^4^, ^5^

Outcomes following NORSE vary widely. The in‐hospital mortality rate of NORSE is approximately 12% in children and even higher in adults, ranging from 9% to 42%.^2^, ^6^, ^7^, ^8^, ^9^, ^10^, ^11^, ^12^, ^13^ Among survivors, prognosis is often poor, with a high prevalence of long‐term cognitive deficits, behavioral disturbances, and epilepsy. Several case series have described variations in outcomes based on factors such as age, SE etiology, duration of RSE, and acute medical complications.^2^, ^7^, ^8^, ^10^ However, there is considerable heterogeneity in how long‐term outcomes are assessed across studies.^14^, ^15^ A recent review analyzing data from 280 adults and 587 children reported that most patients were unable to return to their baseline level of functioning.^16^ Although seizure outcomes have been a primary focus, long‐term cognitive outcomes have been assessed more frequently in children than in adults, but not frequently beyond 36 months.

Recently, the international NORSE Institute long‐term outcome working group published research priorities and suggested focusing on eight key long‐term outcome domains, including quality of life (QOL).^14^ Interviews with patients and caregivers revealed that NORSE has significant life‐altering impacts affecting not only the patients themselves but also their caregivers.^17^ Key challenges include persistent seizures, mental and behavioral health issues, and changes in relationships with family and friends, all of which contribute to reduced independence, greater social difficulties, and lower QOL.^17^ Similarly, a socioeconomic study found that patients with a history of SE were less likely to live independently at home after SE compared to those with drug‐resistant epilepsy or seizure‐free individuals.^18^ Additionally, major depression was frequently observed in patients with SE, who reported a lower QOL compared to patients with epilepsy in seizure remission.^18^

Several tools have been validated for assessing QOL in patients with epilepsy, including the Neuro‐QOL, a system of patient‐reported outcome measures used in neurology clinical research.^19^ This tool has been validated in both adults and children with epilepsy.^20^, ^21^ In this study, we used the Neuro‐QOL to assess physical, mental, and social health in adults and children with NORSE. We aimed to investigate long‐term QOL outcomes and their trajectories over time to help guide psychological and social support strategies for patients and caregivers recovering from NORSE.

## 2 MATERIALS AND METHODS

### 2.1 Study design, settings, and participants

This study was approved by the Yale University ethics committee (NORSE, IRB #1511016840 and NORSE/FIRES biorepository, IRB #200031611). Patients >6 years old hospitalized with NORSE (*n* = 47, including eight children and 39 adults) were prospectively enrolled from 19 centers across the United States (*n* = 12 centers), Canada (*n* = 2), Belgium (*n* = 2), France (*n* = 2), and South Korea (*n* = 1). Patients were enrolled from December 2017 to January 2025 in the NORSE multicenter study (for which Yale is the coordinating center) and the NORSE/FIRES biorepository at Yale University. Patients were included in this study if they completed the Neuro‐QOL at least once at 3–6 months, 12 months, 24 months, or ≥36 months after SE ended. Caregivers were instructed to work with patients to complete the questionnaire.

### 2.2 Data collection

Clinical data were entered into a study‐specific secure REDCap database. Prespecified variables included demographics, clinical parameters (such as intensive care unit [ICU] and SE duration), SE presentation characteristics, including prodromal symptoms, and the list of the treatments received during the ICU stay and during follow‐up, including antiseizure medications (ASMs), continuous intravenous anesthetic drugs, and immunotherapies. Patients' functional outcomes were assessed at discharge and at each follow‐up time point using the Glasgow Outcome Scale–Extended (GOS‐E), ranging from 1 (death) to 8 (upper good recovery). Patients who experienced at least two unprovoked post‐NORSE seizures were diagnosed with post‐NORSE epilepsy.

### 2.3 Neuro‐QOL impairment score

The self‐reported Neuro‐QOL scales were used to assess physical, mental, and social health in adults (13 items, 102 questions) and children aged 6–17 years (eight items, 66 questions; Supplementary Material 1 in Data S1). The questions were scored from 1 (i.e., least impairment) to 5 (i.e., most impairment). Traditionally, a higher score for some domains, such as anxiety, indicates worse outcome, whereas a higher score for other domains, such as communication, indicates better outcome. To more easily identify trends across various domains, we reversed some of the raw Neuro‐QOL scores to ensure that higher scores always indicated more impairment (poorer outcome). For example, a response on the Communication form indicating no difficulty communicating is traditionally scored as a 5 on a 1–5 scale, whereas we revised this so that our scoring system represents no difficulty communicating as 1 (least impairment). Therefore, the question “How much difficulty do you currently have writing notes to yourself, such as appointments or ‘to do’ lists?” was scored as 5 if the patient answered “cannot do” and scored at 1 if the patient answered “none.” In contrast, for the form “anxiety”, the statement “In the past 7, I felt uneasy” was scored 5 for “always” and 1 for “never.”

Total QOL impairment (QOL‐I) scores for each item were calculated by summing item scores, dividing by the maximum possible scores (to adjust for questions lacking a response), and converting to percentages (Figure S1). We calculated the relative change in each QOL‐I subcategory for each patient and the total impairment QOL‐I score over time (i.e., 3–6 to 12, 3–6 to 24, and 12 to 24 months).

### 2.4 *T*‐score evaluation

Whereas QOL‐I scores allow for individualized tracking of impairment over time, *T*‐scores contextualize the severity of impairment against reference populations. By interpreting both together, we gain complementary insights. We calculated *T*‐scores for each item using the standard scoring system provided by the Health Measures Scoring Service.^22^ In line with procedures used for control populations, the score conversions (i.e., reversals of ordering) applied when calculating QOL‐I scores were not performed when computing *T*‐scores. On the *T*‐score scale, a value of 50 represents the mean of the reference population. A score of 40 indicates 1 SD below the mean, and a score of 60 represents 1 SD above the mean. A higher *T*‐score for participation or satisfaction with social roles, positive affect, and cognitive function highlights an improvement, whereas a higher *T*‐score represents a worsening for emotional behavior and dyscontrol, fatigue, sleep disturbances, stigma, anger, anxiety, depression, and pain.

For adults, comparisons were made with the general population for participation and satisfaction with social roles and activities, and positive affect and well‐being. In contrast, clinical neurology populations—including individuals with stroke, epilepsy, multiple sclerosis, Parkinson disease, and amyotrophic lateral sclerosis—were used as reference groups for fatigue, sleep disturbances, stigma, and emotional and behavioral dyscontrol.^22^ *T*‐scores were not calculated for the other domains due to the lack of suitable reference populations.

For children, general population norms were used for all domains, except for pain and stigma, for which reference data from patients with muscular dystrophy and epilepsy were applied.^22^

### 2.5 Statistical analysis

Analyses were performed with R (v.2023.03.1). The data are represented as median [Q1–Q3] for all values except *T*‐scores, for which we report mean ± SD. We performed Kruskal–Wallis tests to evaluate the impact of time points on QOL‐I scores. Post hoc comparisons were performed by using Dunn tests when appropriate. The evolution over time for specific individuals was compared by using the Wilcoxon matched‐pairs signed‐rank test. We used the Friedman test when comparing more than two time points. Post hoc pairwise comparisons were conducted using Wilcoxon signed‐rank tests. Correlations among QOL‐I scores and clinical data were evaluated by calculating Spearman rho values and their level of significance. All statistical tests were two‐sided, with a type I error rate of 5%. We did not adjust for multiple comparisons due to the exploratory nature of the study in modest sized cohorts.

## 3 RESULTS

### 3.1 Study participants

Among the 102 patients aged 6 years or older enrolled in the NORSE biorepositories at Yale from December 2017 to January 2025 who survived SE, 47 completed the Neuro‐QOL at least once during their follow‐up, including 39 adults and eight children. Outcomes were assessed within 3–6 months for 37 patients (29 adults and eight children), at 12 months for 29 patients (24 adults and five children), at 24 months for 23 patients (20 adults and three children), and at ≥36 months for nine patients (eight adults and one children). Among them, 30 patients (24 adults and six children) had at least two follow‐ups. Table 1 summarizes the clinical characteristics of the patients. No significant differences were observed in SE‐related clinical parameters, including SE duration, length of ICU stay, treatments received, functional outcomes, or biological markers of liver and renal function, among patients who completed the forms at different time points. The most frequently used continuous anesthetics were propofol (59% adults, 88% children) and midazolam (64% adults, 88% children). A total of 20 different ASMs were used in adults, with the most common being levetiracetam (90%), lacosamide (77%), and valproic acid (51%). In children, 16 different ASMs were used, most frequently levetiracetam (88%), phenobarbital (88%), and valproic acid (75%).

**TABLE 1:** Clinical information of the study cohort.

Characteristic	Adults, total 39 patients				
	Entire cohort, 39 patients	3–6 months, 29 patients	12 months, 24 patients	24 months, 20 patients	36+ months, 8 patients
Age at SE onset, years	32 [22–50]	35 [26–53]	34 [26–52]	36 [26–54]	27 [18–42]
Sex (% male)	11/39 (28%)	7/29 (24%)	5/24 (21%)	4/20 (20%)	4/8 (50%)
FIRES	25/39 (64%)	16/29 (55%)	13/24 (54%)	11/20 (55%)	6/8 (75%)
Cryptogenic	31/39 (79%)	21/29 (72%)	19/24 (79%)	15/20 (75%)	8/8 (100%)
SE duration, days	11 [4–25]	8 [4–18]	11 [5–14]	11 [4–15]	25 [11–41]
ICU duration, days	21 [13–46]	20 [12–36]	21 [14–41]	23 [15–45]	38 [12–55]
Number of CIVADs during the ICU stay	2 [1–3]	2 [1–2]	2 [1–2]	2 [1–2]	2 [1–3]
Number of ASMs during the ICU stay	5 [4–8]	5 [4–7]	5 [4–7]	5 [4–6]	5 [3–7]
Number of immunotherapies during the ICU stay	3 [2–3]	2 [1–3]	2 [1–3]	3 [2–3]	3 [3–3]
Highest AST during the ICU stay, IU/L^a^	74 [45–164]	68 [41–101]	68 [39–97]	71 [44–164]	77 [51–129]
Highest ALT during the ICU stay, IU/L^a^	93 [53–177]	73 [46–177]	82 [51–181]	89 [55–229]	124 [123–243]
Highest creatinine during the ICU stay, mg/dL^a^	.82 [.69–.93]	.78 [.69–.93]	.75 [.68–.93]	.79 [.69–.90]	.90 [.83–1.01]
Highest BUN during the ICU stay, mg/dL^a^	28.1 [20.8–44]	27.6 [20.3–39]	27 [20–40]	28.1 [21.8–60]	50.7 [43.2–58.1]
GOS‐E at ICU discharge^b^	3 [3–4]	3 [3–4]	3 [3–4]	3 [3–4]	3 [3–4]
Epilepsy post‐NORSE	NA	11/29 (38%)	14/24 (58%)	10/20 (50%)	7/8 (88%)
GOS‐E during follow‐up^b^	NA	6 [4–6]	6 [4–7]	6 [5–8]	6 [6–7]
Number of ASMs during follow‐up	NA	2 [1–4]	3 [1–4]	3 [0–4]	4 [3–4]

	Children, total 8 patients				
	Entire cohort, 8 patients	3–6 months, 8 patients	12 months, 5 patients	24 months, 3 patients	36+ months, 1 patient
Age at SE onset, years	8 [7–13]	8 [7–13]	12 [7–15]	9 [8–12]	6 [NA]
Sex (% male)	5/8 (63%)	5/8 (63%)	4/5 (80%)	3/3 (100%)	1/1 (100%)
FIRES	6/8 (75%)	6/8 (75%)	4/5 (80%)	2/3 (67%)	1/1 (100%)
Cryptogenic	7/8 (88%)	7/8 (88%)	5/5 (100%)	3/3 (100%)	1/1 (100%)
SE duration, days	9 [3–24]	9 [3–24]	11 [7–17]	3 [2–7]	3 [NA]
ICU duration, days	31 [12–50]	31 [12–50]	33 [28–50]	8 [5–18]	2 [NA]
Number of CIVADs during the ICU stay	3 [2–3]	3 [2–3]	3 [2–3]	2 [2–2]	2 [NA]
Number of ASMs during the ICU stay	7 [6–7]	7 [6–7]	6 [5–8]	5 [4–6]	2 [NA]
Number of immunotherapies during the ICU stay	3 [2–4]	3 [2–4]	3 [2–5]	0 [0–1]	0 [NA]
Highest AST during the ICU stay, IU/L^a^	98 [53–112]	98 [53–112]	98 [72–142]	98 [72–105]	46 [NA]
Highest ALT during the ICU stay, IU/L^a^	39 [31–124]	39 [31–124]	124 [75–179]	39 [32–82]	25 [NA]
Highest creatinine during the ICU stay, mg/dL^a^	.47 [.44–.60]	.47 [.44–.60]	.60 [.52–.66]	.47 [.46–.60]	.44 [NA]
Highest BUN during the ICU stay, mg/dL^a^	20 [19–29]	20 [19–29]	29 [21–29]	20 [16–25]	12 [NA]
GOS‐E at ICU discharge^b^	6 [5–7]	6 [5–7]	5 [5–6]	7 [7–8]	8 [NA]
Epilepsy post‐NORSE	2/8 (25%)	2/8 (25%)	2/5 (40%)	0/3 (0%)	0/1 (0%)
GOS‐E during follow‐up^b^	NA	7 [7–8]	7 [7–7]	8 [7–8]	8 [NA]
Number of ASMs during follow‐up	NA	3 [2–4]	2 [2–3]	0 [0–2]	0 [NA]

*Note*: Continuous variables are represented as median [Q1–Q3].

Abbreviations: ALT, alanine aminotransferase; ASM, antiseizure medication; AST, aspartate aminotransferase; BUN, blood urea nitrogen; CIVAD, continuous intravenous anesthetic drug; FIRES, febrile infection‐related epilepsy syndrome; GOS‐E, Glasgow Outcome Scale–Extended; ICU, intensive care unit; NA, not applicable; NORSE, new onset refractory status epilepticus; SE, status epilepticus.

^a^ Biological information was available for 33 of the 39 adults and five of the eight children.

^b^ Functional outcome was evaluated with the GOS‐E ranging from 1 (death) to 8 (upper good recovery) for adults and children.

Ninety percent of adults received at least one immunotherapy, and 38% received at least one second‐line immunotherapy (rituximab 28%, anakinra and tocilizumab 8%). Among children, 75% received immunotherapy, and 50% received at least one second‐line immunotherapy (anakinra 38%, tocilizumab 25%, and rituximab 12.5%).

### 3.2 QOL‐I scores

In adults, overall impairment, as measured by the total QOL‐I score, showed a gradual improvement, with median scores decreasing (improving) from 44.1% at 3–6 months to 37.6% at ≥36 months (Table 2). Fatigue and satisfaction with social roles and activities were the most affected functions at 3–6 and 12 months. By 24 months and beyond, fatigue remained the most impaired domain; however, satisfaction with social roles and activities showed improvement, whereas anxiety and cognitive difficulties became more prominent. Impairment in upper and lower extremity function remained stable throughout the study period. When considering all patients in an unpaired analysis, no statistically significant differences were observed across time points for any domains.

**TABLE 2:** Quality of life impairment scores for adults and children.

	Adults, total 39 patients			
	3–6 months, 29 patients	12 months, 24 patients	24 months, 20 patients	36+ months, 8 patients
Communication	32 [20–44]	28 [20–36]	28 [24–42]	32 [24–45]
Ability to participate in social roles and activities	42.5 [30–62.5]	42.5 [28.9–55.6]	32.5 [24.3–43.1]	36.3 [24.4–60.6]
Satisfaction with social roles and activities	53.8 [37.5–75]	60 [40.6–70]	45 [24.4–68.1]	42.5 [29.4–63.1]
Anxiety	42.5 [27.5–55]	47.5 [35–56.9]	45 [37.5–60.6]	52.5 [43.8–57.5]
Depression	35 [22.5–57.5]	41.3 [24.4–54.4]	40 [29.4–45]	37.5 [23.8–41.3]
Emotional and behavioral dyscontrol	40 [26.3–56.3]	37.5 [26.3–53.8]	37.5 [26.3–48.1]	37.5 [28.8–45]
Fatigue	55 [42.5–62.5]	60 [36.3–73.8]	53.8 [41.9–70.6]	57.5 [55.6–64.3]
Upper extremity function	20 [20–25]	20 [20–22.5]	20 [20–23.1]	20 [20–25]
Lower extremity function	27.5 [20–32.5]	22.5 [20–40]	20 [20–33.1]	21.3 [20–26.3]
Positive affect and well‐being	46.7 [32.2–60]	42.2 [35.6–64.4]	41.1 [34.4–60]	32.2 [21.7–56.3]
Sleep disturbance	40 [30–50]	45 [37.5–54.6]	43.8 [38.8–53.1]	41.3 [34.4–48.8]
Stigma	30 [23.8–38.8]	32.5 [22.9–42.5]	35 [25–42.5]	52.5 [29.4–63.1]
Cognition function	47.5 [35–57.5]	48.8 [35.6–63.5]	53.8 [39.4–60]	55 [49.4–88.8]
Total impairment score (QOL‐I)	44.1 [32.4–51.7]	41.1 [35–52.5]	38.7 [34.6–51.3]	37.6 [34.2–55.6]

	Children, total 8 patients			
	3–6 months, 8 patients	12 months, 5 patients	24 months, 3 patients	36+ months, 1 patient
Social relations–interaction with peers	47.5 [37–55.6]	40 [35–50]	35 [31.3–43.8]	27.5 [NA]
Anxiety	41.3 [29.4–48.8]	32.5 [30–40]	52.5 [41.3–57.5]	27.5 [NA]
Depression	32.5 [20–45]	30 [25–32.5]	32.5 [27.5–41.3]	30 [NA]
Anger	32.5 [21.9–58.1]	37.5 [20–55]	55 [42.5–56.3]	37.5 [NA]
Pain	20 [20–23]	24 [20–26]	24 [23–32]	20 [NA]
Fatigue	33.8 [23.8–41.3]	30 [25–45]	25 [22.5–45]	25 [NA]
Stigma	25 [20–27.5]	25 [20–27.5]	20 [20–27.5]	20 [NA]
Cognitive function	45 [44.4–48.8]	52.5 [45–67.5]	47.5 [46.3–62.5]	35 [NA]
Total impairment score (QOL‐I)	36.8 [29.7–42.9]	33 [30.9–39.4]	33 [31.5–43.8]	27.6 [NA]

*Note*: Data are represented as median [Q1–Q3]. Higher scores indicate more impairment.

Abbreviations: NA, not applicable; QOL‐I, quality of life impairment.

Despite the small sample size in children, the total QOL‐I score demonstrated a gradual improvement over time, with median scores decreasing from 36.8% at 3–6 months to 27.6% at ≥36 months (Table 2). Compared to adults, there was greater heterogeneity in the domains affected. During the first year, social relations and cognitive functions were the most impaired, whereas by 24 months, difficulties related to anxiety and anger became more prominent. As with adults, unpaired analysis showed no statistically significant differences across time points for any domains.

### 3.3 Evolution over time of QOL‐I

Paired analyses were performed to determine whether individual changes over time were statistically significant across time points. In adults, the total QOL‐I score improved between 3–6 and 12 months (22 patients, 45.5% [39.0–55.0] vs. 41.1% [35.4–52.9], *p* = .051), and between 3–6 and 24 months (18 patients, 45.5% [41.2–53.8] vs. 38.7% [35.3–51.5], *p* = .016).

Further analysis revealed improvements in specific domains. Between 3–6 and 12 months, communication improved (36.0% [20.0–48.0] vs. 29.0% [20.0–36.0], *p* = .042). Between 3–6 and 24 months, improvements were observed in participation in social roles and activities (53.8% [40.0–66.3] vs. 36.3% [26.8–44.4], *p* = .018), satisfaction with social roles and activities (57.5% [47.5–85.0] vs. 45.0% [27.5–66.3], *p* = .035), fatigue (57.3% [50.4–71.9] vs. 53.8% [43.1–67.5], *p* = .028), and lower extremity function (30.0% [27.5–36.9] vs. 21.3% [20.0–34.4], *p* = .036). No significant changes were observed between 12 and 24 months for the 18 patients.

Seventeen patients completed the scales at 3–6, 12, and 24 months. Analyses revealed a significant improvement in fatigue (Friedman test, *p* = .034), which was most pronounced between 3–6 and 24 months (*p* = .042).

For children, analysis was limited to those who completed the scale at both 3–6 and 12 months (five patients), and no significant differences were observed across any domains.

Detailed results are presented in Table S1.

### 3.4 Correlation of QOL‐I with biological and clinical data

**FIGURE 1:**
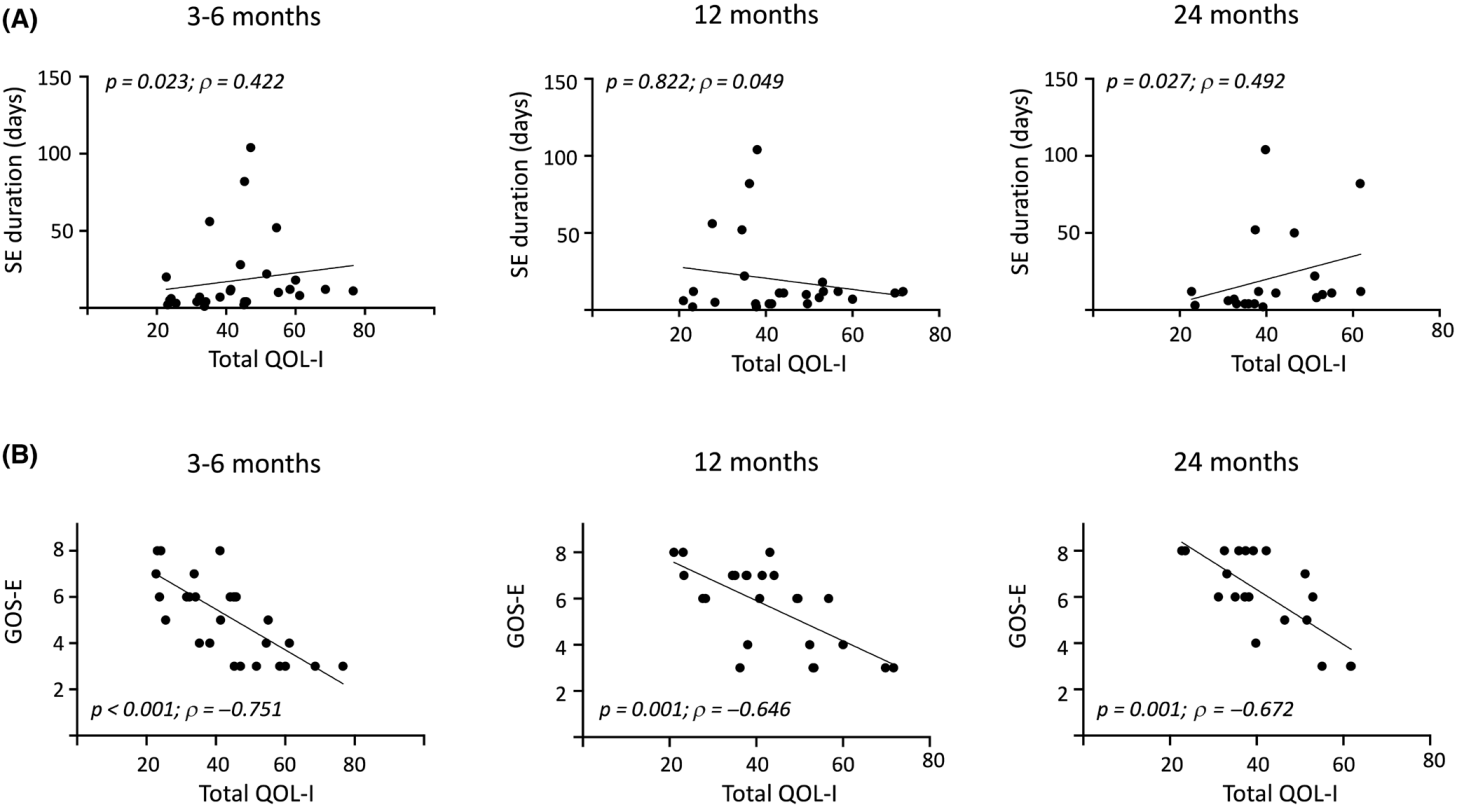
Correlation of clinical features in adults with total quality of life impairment (QOL‐I) score at 3–6, 12, and 24 months. Correlations were evaluated by calculating Spearman rho values and their level of significance. GOS‐E, Glasgow Outcome Scale–Extended; SE, status epilepticus.

Next, we determined whether the QOL‐I correlated with biological and clinical characteristics at the time of SE stay or at follow‐up assessments (Figure 1). Patients with higher blood urea nitrogen levels during the ICU stay had a higher QOL‐I and worse functional outcomes at 3–6 (*ρ* = .450, *p* = .021; and *ρ* = −.431, *p* = .028) and 12 (*ρ* = .528, *p* = .014; and *ρ* = −.518, *p* = .016) months, without significant correlation at 24 months. No significant correlation was observed for hepatic function biomarkers or with creatinine. Adults with longer SE durations experienced worse functional outcomes, as indicated by lower GOS scores at 3–6 months (Spearman ρ = −.617, *p* < .001), 12 months (*ρ* = −.410, *p* = .047), and 24 months (*ρ* = −.427, *p* = .060). Longer SE duration also correlated with greater impairment across several domains at 3–6 months, including communication (*ρ* = .374, *p* = .046), participation in social roles and activities (*ρ* = .398, *p* = .033), depression (*ρ* = .486, *p* = .008), and stigma (*ρ* = .406, *p* = .036). Similarly, longer SE duration was associated with emotional and behavioral disturbances (*ρ* = .476, *p* = .034), fatigue (*ρ* = .500, *p* = = .025), and stigma (*ρ* = .586, *p* = .008) at 24 months. The duration of SE was associated with higher total QOL‐I scores at 3–6 months (*ρ* = .422, *p* = .023) and 24 months (*ρ* = .492, *p* = .027; Figure 1A).

Longer ICU stays were also associated with higher (worse) QOL‐I scores at 24 months (*ρ* = .492, *p* = .028), particularly concerning anxiety (*ρ* = .500, *p* = .025), emotional challenges (*ρ* = .568, *p* = .009), fatigue (*ρ* = .493, *p* = .027), and cognitive function (*ρ* = .459, *p* = .042).

Overall, patients with the most impaired QOL‐I profiles consistently had worse functional outcomes, as measured by the GOS‐E, at all follow‐up points (3–6 months: *ρ* = −.751, *p* < .001; 12 months: *ρ* = −.646, *p* = .001; 24 months: *ρ* = −.672, *p* = .001; Figure 1B). However, these individuals did not have a higher prevalence of epilepsy or greater use of ASMs during follow‐up. Whereas stigma scores showed a borderline correlation with post‐NORSE epilepsy at 3–6 (*p* = .049) and 24 (*p* = .046) months, there were no significant differences in median stigma scores between patients with and without epilepsy.

Notably, patients taking a higher number of ASMs at 3–6 months reported greater depression‐related impairment (*ρ* = .506, *p* = .005), and patients taking a higher number of ASMs at 24 months reported increased stigma (*ρ* = .827, *p* < .001) and cognitive impairments (*ρ* = .515, *p* = .020).

Figure S2 illustrates the correlations at each time point. Correlation analyses were not conducted in children due to the small size of the cohort.

### 3.5 Comparison with reference populations: *T*‐scores

In adults, all but one QOL domain had *T*‐scores within half an SD of the reference population mean (Table 3). Satisfaction with social roles and activities was the only domain with *T*‐scores more than half an SD below the reference mean, indicating greater impairment compared to the reference population. We then assessed the mean change in *T*‐scores over time. Whereas no statistically significant differences were observed between 3–6 and 12 months, there was a significant improvement in participation in social roles (mean change = 5.5, *p* = .02) and a reduction in fatigue (mean change = −2.7, *p* = .021) between 3–6 and 24 months.

**TABLE 3:** *T*‐score evaluation in patients with new onset refractory status epilepticus at the different time points.

Neuro‐QOL short form	3–6 months, mean ± SD	12 months, mean ± SD	24 months, mean ± SD	Chronic, mean ± SD	Mean change (minimum change, maximum change) from 3–6 to 12 months [adults: 22 patients, children: 5 patients]	Paired analysis for the mean change between 3–6 and 12 months	Mean change (minimum change, maximum change) from 3–6 to 24 months [adults: 18 patients, children: 3 patients]	Paired analysis for the mean change between 3–6 and 24 months
Adults								
Participation in social roles^a^	45.3 ± 8.2	46.5 ± 8.4	48.9 ± 8.0	47.3 ± 8.4	2.4 (−8.9, 27.8)	.16	5.5 (−12.4, 19.5)	.**02**
Satisfaction with social roles^a^	44.8 ± 8.0	46.2 ± 7.7	47.0 ± 8.4	43.9 ± 5.7	3.2 (−11.6, 19.4)	.06	4.1 (−12.4, 24.7)	.10
Positive affect^a^	52.1 ± 10.3	52.2 ± 8.8	52.7 ± 7.9	56.3 ± 10.1	1.5 (−23.3, 27.8)	.44	1.5 (−13.9, 27.8)	.49
Emotional behavior and dyscontrol^b^	48.8 ± 11.6	48.6 ± 10.4	47.7 ± 9.6	45.5 ± 9.4	−1.7 (−24.5, 32.4)	.35	−1.0 (−20.3, 16.6)	.67
Fatigue^b^	47.7 ± 8.5	49.6 ± 10.6	48.6 ± 8.2	51.1 ± 6.2	.76 (−15.7, 16.7)	.50	−2.7 (−12.9, 7)	.**021**
Sleep^b^	50.0 ± 9.2	51.2 ± 8.4	51.8 ± 8.8	51.4 ± 6.1	−.79 (−13.1, 13.4)	.67	.15 (−17.4, 17)	>.99
Stigma^b^	49.6 ± 7.0	51.0 ± 7.2	51.6 ± 6.7	55.7 ± 8.7	−.74 (−12.4, 12.7)	.71	−.7 (−8.7, 15.2)	.26
Children								
Anger^a^	49.5 ± 12.8	48.2 ± 12.9	54.9 ± 5.8	NA	−1.7 (−6.8, 4.3)	.38	4.2 (−4.6, 17.9)	NA
Anxiety^a^	51.3 ± 10.3	51.1 ± 5.2	56.8 ± 7.8	NA	−1.2 (−20.7, 9.4)	.81	−.8 (−20.7, 12.9)	NA
Cognitive^a^	45.8 ± 3.1	41.5 ± 6.0	41.3 ± 5.9	NA	−4.6 (−10.7, 1)	.19	−4.6 (−12.8, −.5)	NA
Depression^a^	46.2 ± 9.3	47.1 ± 7.7	48.3 ± 5.5	NA	−1.4 (−11.9, 8.1)	.88	2.2 (−11.9, 11.2)	NA
Social relations^a^	44.4 ± 5.7	48.6 ± 6.9	50.4 ± 6.2	NA	7.5 (−2, 18.3)	.25	6.2 (−2.8, 18.3)	NA
Pain^c^	42.6 ± 7.2	45.6 ± 7.2	48.4 ± 5.4	NA	3.6 (−.2, 10.6)	.25	9.6 (5.8, 16.1)	NA
Stigma^c^	42.6 ± 4.9	43.0 ± 5.6	40.8 ± 6.4	NA	−1.1 (−6.1, 3)	.63	−.6 (−6.1, 4.3)	NA

*Note*: The differences in *T*‐scores over time have been assessed with Wilcoxon matched‐pairs signed‐rank tests.

Abbreviation: NA, not available.

^a^ Items were compared to the general population.

^b^ Items were compared to clinical neurology populations (i.e., stroke, epilepsy, multiple sclerosis, Parkinson disease, amyotrophic lateral sclerosis).

^c^ Items were compared to individuals with muscular dystrophy and epilepsy.

In our cohort of pediatric patients, we once again saw that most domains across all time points had *T*‐scores within half an SD of the reference population mean. In contrast, we found that the mean *T*‐scores were closer to a full SD below the reference population means for cognitive function at 12 and 24 months (41.5 ± 6.0 and 41.3 ± 5.9), suggesting that our cohort had more impaired cognitive function (worse outcome) compared to the reference population. Similarly, the *T*‐scores differed from the control population for pain at 3–6 months (42.6 ± 7.2) and stigma at all time points (42.6 ± 4.9 at 3–6 months, 43.0 ± 5.6 at 12 months, and 40.8 ± 6.4 at 24 months), suggesting less impairment (improved outcome) compared to the reference population. There were no statistically significant differences in the mean change of *T*‐scores between 3–6 and 12 months.

## 4 DISCUSSION

Our study is one of the first multicenter, long‐term prospective studies on neurological QOL outcomes in adults and children following NORSE. We observed an overall improvement in QOL in the first few years following hospital discharge. However, some outcomes remain consistently impaired, and some worsen over time. These findings highlight the need for targeted long‐term support for survivors.

In adults, we observed significant improvements in overall QOL and in several specific QOL domains over time, particularly between 3–6 months and 24 months post‐NORSE. Domains such as fatigue, satisfaction with social roles, communication, and mobility showed notable improvement, suggesting a trajectory of partial neurological recovery or psychosocial adaptation. In case of discrepancies in the domains showing improvement, we prioritized the QOL‐I scores, as they reflect individual patient trajectories without relying on a normative reference. Overall improvements align with prior findings that demonstrated some functional recovery over time in NORSE survivors.^2^, ^12^ Most improvement occurred during the first year, with relatively less change between 12 and 24 months. These results help clarify questions raised regarding the length of observations necessary for long‐term outcome studies of NORSE by indicating that the first 12 months are most critically important for recovery and sustained rehabilitation efforts.^14^ Similar results were observed in prior research focusing on rehabilitative services in patients poststroke.^23^, ^24^ In children, we were not able to find significant patterns of improvement over time, possibly due to the limited sample size. This underscores the importance of extending the long‐term outcome studies in children with NORSE/FIRES to include QOL measures.^25^

Despite overall improvements, certain domains remained persistently impaired in adults. Fatigue was consistently among the most impaired functions across all time points, consistent with literature indicating that fatigue is very common after neurological injury. This etiology of fatigue is multifactorial; it may include the sequelae of the direct neurological damage and secondary factors such as mood disorders, sleep disruption, or side effects of medications.^26^, ^27^ At 24 months, whereas satisfaction with social roles improved, anxiety and cognitive difficulties became more prominent. Longer ICU stays were associated with greater anxiety and cognitive dysfunction, suggesting a link between prolonged critical illness and persistent neuropsychiatric symptoms. Notably, prior studies found that depression and anxiety were reported in 10%–60% of patients admitted to the ICU, with a higher risk of posttraumatic stress disorder for those requiring heavy sedation, as is required for NORSE patients.^28^ The impairments in cognitive function are consistent with prior studies that reported a high prevalence of cognitive impairments in NORSE survivors.^29^ The trend toward increasing prevalence of cognitive concerns over time could suggest a progressive component to the disorder; however, more evidence is needed to draw strong conclusions. The rising prevalence of cognitive concerns over time could also reflect survivors' return to work and increased social responsibilities, where cognitive deficits become more apparent, even though they report greater satisfaction with their social role evolution.^30^ In children, early impairments were mainly in social relations and cognitive function, as in adults. We were not surprised to find that satisfaction with social roles and activities would be impaired in NORSE survivors, given the existing literature about how both acute neurological injury and epilepsy can affect social roles.^31^, ^32^ However, the improvement over time may suggest that survivors can adapt to their new roles and relationships. Prior studies also affirm that most children who survive the acute episode have significant cognitive impairments and learning disabilities.^6^ Over time, anxiety and anger became more prominent, a finding less documented in prior NORSE studies. This highlights that even children who demonstrate good neurological recovery continue to struggle with emotional and psychosocial issues, necessitating a comprehensive, long‐term support system.

We found that clinical severity markers correlated strongly with long‐term outcomes. In adults, longer SE duration was associated with worse communication, stigma, and participation in social roles, perhaps due to an association with increased neurological damage.^33^ Length of ICU stay was correlated with worse outcomes in anxiety, emotional and behavioral dyscontrol, fatigue, and cognitive function. Our findings regarding the relationship between the length of ICU stay and poor outcomes address a gap posed by a previous retrospective study of NORSE in children, which did not demonstrate a relationship between these clinical variables and prognosis.^3^ In addition, consistent with prior literature linking polytherapy to reduced QOL,^34^, ^35^, ^36^ the use of a higher number of ASMs at follow‐up visits in our study correlated with greater depression, cognitive impairment, and stigma.

We anticipate that early, intensive multidisciplinary rehabilitation, particularly within the first year after NORSE, may help to improve long‐term outcomes. Rehabilitation strategies should not only address medical needs but also provide psychological support, and future research should address the role of psychiatric counseling and therapeutics in recovery. Social support is also instrumental in helping to reintegrate survivors into society by facilitating a smoother transition into post‐NORSE life^37^ and should be addressed proactively by clinicians.^38^ Although the observed improvement over time may be used to encourage family members to remain hopeful of a more favorable outcome, we still have limited information regarding outcomes beyond 36 months.

Limitations of our study include the modest cohort size, especially among children, incomplete longitudinal data, and potential selection bias toward survivors with better recovery who were able to complete the Neuro‐QOL. Although all patients were evaluated for the occurrence of spontaneous seizures following SE resolution, data on recurrent episodes of SE or rehospitalizations due to seizure recurrence or clinical worsening during follow‐up were not available. This limitation is partly due to the multicenter design of the study, which made consistent longitudinal data collection across sites challenging and may restrict the comprehensive assessment of long‐term seizure burden. In addition, caregiver‐assisted reporting may also introduce bias and affect the reliability of self‐reported QOL outcomes.^39^, ^40^ Lastly, whether the changes we report are large enough to be clinically meaningful is difficult to determine. Currently, no minimal clinically important difference (MCID) or reliable change index has been established for the Neuro‐QOL in the context of NORSE. This limitation makes it challenging to determine whether the statistically significant improvements observed are also clinically meaningful.^19^ Nevertheless, the magnitude and consistency of improvement observed across domains such as fatigue, participation in social roles, and satisfaction with social roles may reflect clinically meaningful changes.

Future research should focus on larger, longitudinal pediatric cohorts and comparative studies of NORSE survivors to other epilepsy and critical illness populations. When funding allows, these studies should measure outcomes beyond 36 months to determine whether the observed trends continue in the following years. Furthermore, these future studies will help clarify which dimensions of QOL impairment are due to the comorbid post‐NORSE epilepsy and which are due to the damage sustained during the initial episode, either from underlying brain injury or the effects of prolonged SE itself. Expanding our understanding of long‐term psychosocial recovery trajectories will be critical in designing comprehensive care models to support this vulnerable population. Future studies should aim to establish MCIDs for Neuro‐QoL measures in NORSE, using a combination of anchor‐based methods (e.g., functional outcome scales) and distribution‐based approaches, as has been done for the Quality of Life in Childhood Epilepsy Questionnaire.^41^ Ideally, both patient‐reported and clinician‐assessed indicators of meaningful change should be integrated.

## 5 CONCLUSIONS

Our study provides important insights into the psychosocial, physical, and cognitive sequelae faced by survivors in the years following the acute NORSE episode. Although QOL in NORSE survivors tends to improve over time, challenges persist across multiple domains. Acute disease severity markers, including duration of both SE and ICU stay, strongly predict poorer long‐term outcomes, underscoring the critical importance of early interventions. These results clarify the broad and lasting impact of NORSE while offering reassurance that survivors can expect improvements in QOL over the first few years following the acute episode. Finally, our findings highlight the need for an early, holistic, multidisciplinary approach to post‐NORSE recovery, addressing medical, psychological, and social needs.

## AUTHOR CONTRIBUTIONS

*Conception and design of the study:* Matthew D. Gruen, Lawrence J. Hirsch, and Aurélie Hanin. *Acquisition and analysis of the data:* Matthew D. Gruen, Margaret T. Gopaul, Anthony D. Jimenez, Ayush Batra, Leah J. Blank, Charlotte Damien, Gregory S. Day, Krista Eschbach, Elizabeth E. Gerard, Teneille E. Gofton, Stephen T. Hantus, Nathalie Jette, Amy Jongeling, Peter Kang, Karnig Kazazian, Marissa Kellogg, Minjee Kim, Bahar Madani, Mikaela Morales, Vineet Punia, Claude Steriade, Aaron Struck, Olga Taraschenko, Nathan Torcida, Mark S. Wainwright, Ji Yeoun Yoo, Nicolas Gaspard, Nora Wong, Lawrence J. Hirsch, and Aurélie Hanin. *Drafting a significant proportion of the manuscript or figures:* Matthew D. Gruen and Aurélie Hanin.

## CONFLICT OF INTEREST STATEMENT

L.J.H. has received support from Yale University for investigator‐initiated studies from the Daniel Raymond Wong Neurology Research Fund and the NORSE/FIRES Biorepository Research Fund, both at Yale. A.H. has received postdoctoral grants from the Paratonnerre Association, the Swebilius Foundation, the Servier Institute, and the Philippe Foundation for NORSE‐related research. N.W. is the executive director of the NORSE Institute. L.J.H. and N.G. are cochairs of the Medical and Scientific Advisory Board of the NORSE Institute. M.T.G., K.E., T.E.G., M.Ke., O.T., and A.H. are members of the Medical and Scientific Advisory Board of the NORSE Institute. K.E. has received funding from the friends and family of Finn Mussetter for NORSE and FIRES research. T.E.G. has received funding from the Robert N. Kohn NORSE Family Registry Memorial Research Fund for NORSE and FIRES Research. The other authors report no competing interests. We confirm that we have read the Journal's position on issues involved in ethical publication and affirm that this report is consistent with those guidelines.

## Supporting information


Table S1.

Figure S1.

Figure S2.

Data S1.

## Data Availability

Anonymized data will be made available by request from any qualified investigator.

## References

[1] Hirsch LJ , Gaspard N , van Baalen A , Nabbout R , Demeret S , Loddenkemper T , et al. Proposed consensus definitions for new‐onset refractory status epilepticus (NORSE), febrile infection‐related epilepsy syndrome (FIRES), and related conditions. Epilepsia. 2018;59(4):739–744. 29399791 10.1111/epi.14016

[2] Gaspard N , Foreman BP , Alvarez V , Cabrera Kang C , Probasco JC , Jongeling AC , et al. New‐onset refractory status epilepticus: etiology, clinical features, and outcome. Neurology. 2015;85(18):1604–1613. 26296517 10.1212/WNL.0000000000001940 PMC4642147

[3] Wu J , Lan X , Yan L , Hu Y , Hong S , Jiang L , et al. A retrospective study of 92 children with new‐onset refractory status epilepticus. Epilepsy Behav. 2021;125:108413. 34794014 10.1016/j.yebeh.2021.108413

[4] deCampo D , Xian J , Karlin A , Sullivan KR , Ruggiero SM , Galer P , et al. Investigating the genetic contribution in febrile infection‐related epilepsy syndrome and refractory status epilepticus. Front Neurol. 2023;14:1161161. 37077567 10.3389/fneur.2023.1161161 PMC10106651

[5] Hanin A , Jimenez AD , Gopaul M , Asbell H , Aydemir S , Basha MM , et al. Trends in management of patients with new‐onset refractory status epilepticus (NORSE) from 2016 to 2023: an interim analysis. Epilepsia. 2024;65(8):e148–e155. 38837761 10.1111/epi.18014

[6] Kramer U , Chi C‐S , Lin K‐L , Specchio N , Sahin M , Olson H , et al. Febrile infection‐related epilepsy syndrome (FIRES): pathogenesis, treatment, and outcome: a multicenter study on 77 children. Epilepsia. 2011;52(11):1956–1965. 21883180 10.1111/j.1528-1167.2011.03250.x

[7] Matthews E , Alkhachroum A , Massad N , Letchinger R , Doyle K , Claassen J , et al. New‐onset super‐refractory status epilepticus: a case series of 26 patients. Neurology. 2020;95(16):e2280–e2285. 32943479 10.1212/WNL.0000000000010787 PMC7713780

[8] Jayalakshmi S , Vooturi S , Sahu S , Yada PK , Mohandas S . Causes and outcomes of new onset status epilepticus and predictors of refractoriness to therapy. J Clin Neurosci. 2016;26:89–94. 26822381 10.1016/j.jocn.2015.06.032

[9] Aurangzeb S , Prisco L , Adcock J , Speirs M , Raby S , Westbrook J , et al. New‐onset super refractory status epilepticus: a case‐series. Seizure. 2020;75:174–184. 31757748 10.1016/j.seizure.2019.10.005

[10] Gugger JJ , Husari K , Probasco JC , Cervenka MC . New‐onset refractory status epilepticus: a retrospective cohort study. Seizure. 2020;74:41–48. 31830676 10.1016/j.seizure.2019.12.002

[11] Shi X , Wang Y , Wang X , Kang X , Yang F , Yuan F , et al. Long‐term outcomes of adult cryptogenic febrile infection‐related epilepsy syndrome (FIRES). Front Neurol. 2022;13:1081388. 36686522 10.3389/fneur.2022.1081388 PMC9848432

[12] Stretti F , Bögli SY , Casagrande F , Eisele A , Galovic M , Keller E , et al. Long‐term outcome in new onset refractory status epilepticus: a retrospective study. Crit Care. 2024;28(1):72. 38475798 10.1186/s13054-024-04858-7 PMC10935909

[13] Werbaneth K , Mausolf M , Seliger J , Le S . A retrospective cohort study of new‐onset refractory status epilepticus (NORSE): clinical features, timing of immunotherapy and outcomes. Epileptic Disord. 2022;24(5):867–876. 35892128 10.1684/epd.2022.1466

[14] Espino PH , Eschbach K , Blank LJ , Cervenka MC , Muscal E , Farias‐Moeller R , et al. New onset refractory status epilepticus: long‐term outcomes beyond seizures. Epilepsia. 2025;66:988–1005. 39825688 10.1111/epi.18267 PMC11997932

[15] Kazazian K , Gaspard N , Hirsch LJ , Kellogg M , Hocker SE , Wong N , et al. Age‐associated differences in FIRES: characterizing prodromal presentation and long‐term outcomes via the web‐based NORSE/FIRES family registry. Epilepsia. 2025;66(3):e35–e40. 39804054 10.1111/epi.18260 PMC11908657

[16] Taraschenko O , Pavuluri S , Schmidt CM , Pulluru YR , Gupta N . Seizure burden and neuropsychological outcomes of new‐onset refractory status epilepticus: systematic review. Front Neurol. 2023;14:1095061. 36761344 10.3389/fneur.2023.1095061 PMC9902772

[17] Eschbach K , Reedy J , Gofton T , Gopaul M , Farias‐Moeller R , Kellogg M , et al. Navigating life after new‐onset refractory status epilepticus (NORSE) and febrile infection‐related epilepsy syndrome (FIRES): insights from caregiver and patient interviews. Epilepsy Behav. 2025;163:110236. 39740258 10.1016/j.yebeh.2024.110236

[18] Kortland L‐M , Knake S , von Podewils F , Rosenow F , Strzelczyk A . Socioeconomic outcome and quality of life in adults after status epilepticus: a multicenter, longitudinal, matched case‐control analysis from Germany. Front Neurol. 2017;8:507. 29018404 10.3389/fneur.2017.00507 PMC5622933

[19] Cella D , Lai J‐S , Nowinski CJ , Victorson D , Peterman A , Miller D , et al. Neuro‐QOL. Neurology. 2012;78(23):1860–1867. 22573626 10.1212/WNL.0b013e318258f744 PMC3369516

[20] Victorson D , Cavazos JE , Holmes GL , Reder AT , Wojna V , Nowinski C , et al. Validity of the neurology quality‐of‐life (neuro‐QoL) measurement system in adult epilepsy. Epilepsy Behav. 2014;31:77–84. 24361767 10.1016/j.yebeh.2013.11.008 PMC3970783

[21] Lai J‐S , Nowinski CJ , Zelko F , Wortman K , Burns J , Nordli DR , et al. Validation of the neuro‐QoL measurement system in children with epilepsy. Epilepsy Behav. 2015;46:209–214. 25862469 10.1016/j.yebeh.2015.02.038 PMC4458416

[22] Neuro‐QOL_Scoring_Manual_26April2021_FINAL.pdf. [cited 2025]. Available from: https://www.healthmeasures.net/images/neuro_qol/User_and_scoring_guides/Neuro‐QOL_Scoring_Manual_26April2021_FINAL.pdf

[23] Stroke rehabilitation – Lancet. [cited 2025]. Available from: https://www.thelancet.com/journals/lancet/article/PIIS0140‐6736(11)60325‐5/abstract

[24] Aziz NA , Leonardi‐Bee J , Phillips M , Gladman JRF , Legg L , Walker MF . Therapy‐based rehabilitation services for patients living at home more than one year after stroke. Cochrane Database Syst Rev. 2008;2008(2):CD005952. 18425928 10.1002/14651858.CD005952.pub2 PMC6464721

[25] Shrestha A , Wood EL , Berrios‐Siervo G , Stredny CM , Boyer K , Vega C , et al. Long‐term neuropsychological outcomes in children with febrile infection‐related epilepsy syndrome (FIRES) treated with anakinra. Front Neurol. 2023;14:1100551. 36970506 10.3389/fneur.2023.1100551 PMC10030614

[26] Kwon O‐Y , Ahn HS , Kim HJ . Fatigue in epilepsy: a systematic review and meta‐analysis. Seizure. 2017;45:151–159. 28063374 10.1016/j.seizure.2016.11.006

[27] Penner I‐K , Paul F . Fatigue as a symptom or comorbidity of neurological diseases. Nat Rev Neurol. 2017;13(11):662–675. 29027539 10.1038/nrneurol.2017.117

[28] Mulkey MA , Beacham P , McCormick MA , Everhart DE , Khan B . Minimizing post–intensive care syndrome to improve outcomes for intensive care unit survivors. Crit Care Nurse. 2022;42(4):68–73. 10.4037/ccn2022374 PMC10350342 35908764

[29] Costello DJ , Matthews E , Aurangzeb S , Doran E , Stack J , Wesselingh R , et al. Clinical outcomes among initial survivors of cryptogenic new‐onset refractory status epilepsy (NORSE). Epilepsia. 2024;65(6):1581–1588. 38498313 10.1111/epi.17950

[30] McPeake J , Mikkelsen ME , Quasim T , Hibbert E , Cannon P , Shaw M , et al. Return to employment after critical illness and its association with psychosocial outcomes. A systematic review and meta‐analysis. Ann ATS. 2019;16(10):1304–1311. 10.1513/AnnalsATS.201903-248OC 31184500

[31] Boden‐Albala B , Litwak E , Elkind MSV , Rundek T , Sacco RL . Social isolation and outcomes post stroke. Neurology. 2005;64(11):1888–1892. 15955939 10.1212/01.WNL.0000163510.79351.AF

[32] Steiger BK , Jokeit H . Why epilepsy challenges social life. Seizure. 2017;44:194–198. 27756511 10.1016/j.seizure.2016.09.008

[33] Trinka E , Leitinger M . Management of Status Epilepticus, refractory status epilepticus, and super‐refractory status epilepticus. Continuum (Minneap Minn). 2022;28(2):559–602. 35393970 10.1212/CON.0000000000001103

[34] Kwan P , Brodie MJ . Neuropsychological effects of epilepsy and antiepileptic drugs. Lancet. 2001;357(9251):216–222. 11213111 10.1016/S0140-6736(00)03600-X

[35] Strzelczyk A , Aledo‐Serrano A , Coppola A , Didelot A , Bates E , Sainz‐Fuertes R , et al. The impact of epilepsy on quality of life: findings from a European survey. Epilepsy Behav. 2023;142:109179. 37058861 10.1016/j.yebeh.2023.109179

[36] Hakami T . Efficacy and tolerability of antiseizure drugs. Ther Adv Neurol Disord. 2021;14:17562864211037430. 34603506 10.1177/17562864211037430 PMC8481725

[37] Batchos E , Easton A , Haak C , Ditchman N . Social factors predictive of social integration for adults with brain injury. Disabil Rehabil. 2018;40(17):2062–2069. 28521554 10.1080/09638288.2017.1326175

[38] Greenwald BD , Harris KA , Ayyala H , Gordon DJ . Community reintegration after traumatic brain injury. Phys Med Rehabil Clin N Am. 2024;35(3):637–650. 38945656 10.1016/j.pmr.2024.02.012

[39] Sebring K , Shattuck J , Berk J , Boersma I , Sillau S , Kluger BM . Assessing the validity of proxy caregiver reporting for potential palliative care outcome measures in Parkinson's disease. Palliat Med. 2018;32(9):1522–1528. 30015552 10.1177/0269216318785830

[40] Costello MM , Judge C , Reddin C , Rangarajan S , Langhorne P , Zhang H , et al. Role of proxy respondents in international stroke research: experience of the INTERSTROKE study. Neuroepidemiology. 2022;56(5):355–364. 35817005 10.1159/000525510

[41] Leda M , Puka K , Bax K , Gagnier JJ , Tassiopoulos K , Speechley KN . Establishing the minimum clinically important difference of the quality of life in childhood epilepsy questionnaire. Epilepsia. 2024;65(12):3536–3544. 39382454 10.1111/epi.18140 PMC11647425

