# Supplementary material for: Quality of life over time after new onset refractory status epilepticus
Source: Epilepsia. 2025 Sep 13;67(1):328–40. doi: 10.1111/epi.18635 (PMC12893261; doi:10.1111/epi.18635)
Supplement: Supplementary file 1 — Table S1. [file EPI-67-328-s004.docx]

|  | **Adults** | | | | | | | | | |
| --- | --- | --- | --- | --- | --- | --- | --- | --- | --- | --- |
|  | **3-6 versus 12 months**  **(22 patients)** | | | **3-6 versus 24 months**  **(18 patients)** | | | **12 versus 24 months**  **(18 patients)** | | | |
|  | 3-6 months | 12 months | p-value | 3-6 months | 24 months | p-value | 12 months | 24 months | p-value |  |
| Communication | 36.0 [28.0] | 29.0 [16.0] | **0.042** | 36.0 [25.0] | 28.0 [20.0] | 0.31 | 29.0 [16.0] | 28.0 [20.0] | 0.44 |  |
| Ability to participate in social roles and activities | 53.8 [27.1] | 42.5 [26.9] | 0.12 | 53.8 [26.3] | 36.3 [17.6] | **0.018** | 42.5 [30.0] | 36.3 [17.6] | 0.12 |  |
| Satisfaction with social roles and activities | 60.0 [37.5] | 60.0 [35.0] | 0.076 | 57.5 [37.5] | 45.0 [38.8] | **0.035** | 60.0 [33.1] | 40.0 [43.1] | 0.11 |  |
| Anxiety | 45.0 [16.9] | 50.0 [22.5] | 0.79 | 45.0 [16.9] | 50.0 [23.8] | >0.99 | 51.3 [26.3] | 50.0 [23.8] | 0.42 |  |
| Depression | 42.5 [31.9] | 46.3 [33.1] | 0.32 | 43.8 [31.9] | 40.0 [13.8] | 0.11 | 48.8 [30.0] | 40.0 [13.8] | 0.070 |  |
| Emotional and behavioral dyscontrol | 41.3 [30.0] | 37.5 [25.0] | 0.15 | 41.3 [25.6] | 38.8 [20.6] | 0.67 | 37.5 [25.6] | 38.8 [20.6] | 0.83 |  |
| Fatigue | 56.1 [20.6] | 60.0 [30.0] | 0.62 | 57.3 [21.5] | 53.8 [24.4] | **0.028** | 64.0 [20.0] | 53.8 [24.4] | 0.28 |  |
| Upper extremity function | 20.0 [10.0] | 20.0 [5.0] | 0.11 | 20.0 [6.88] | 20.0 [4.38] | 0.17 | 20.0 [3.75] | 20.0 [4.38] | 0.31 |  |
| Lower extremity function | 27.5 [13.8] | 25.0 [20.0] | 0.43 | 30.0 [9.38] | 21.3 [14.4] | **0.036** | 23.8 [18.1] | 21.3 [14.4] | 0.54 |  |
| Positive affect and well-being | 47.8 [25.6] | 42.2 [24.4] | 0.28 | 47.8 [26.7] | 43.3 [20.0] | 0.50 | 42.2 [24.4] | 43.3 [20.0] | 0.46 |  |
| Sleep disturbance | 40.0 [20.0] | 45.0 [16.8] | 0.90 | 42.5 [20.6] | 43.8 [14.4] | 0.79 | 42.5 [15.7] | 43.8 [14.4] | 0.80 |  |
| Stigma | 33.8 [11.9] | 32.5 [19.2] | 0.82 | 35.0 [10.0] | 32.5 [15.0] | 0.38 | 32.5 [20.0] | 35.0 [17.5] | 0.57 |  |
| Cognition function | 51.3 [23.8] | 48.8 [26.9] | 0.60 | 52.5 [22.5] | 53.8 [16.7] | 0.48 | 51.3 [25.0] | 53.8 [16.7] | 0.92 |  |
| Total impairment score (QOL-I) | 45.5 [16.0] | 41.1 [17.5] | 0.051 | 45.5 [12.6] | 38.7 [16.2] | **0.016** | 41.1 [15.1] | 38.7 [16.2] | 0.42 |  |
|  | **Children** | | | | | | | | | |
|  | 3-6 versus 12 months  (5 patients) | | | | | | | | | |
|  | 3-6 months | | | 12 months | | | p-value | | |  |
| Social relations – interaction with peers | 53.8 [11.3] | | | 42.5 [18.8] | | | 0.25 | | |  |
| Anxiety | 40.0 [15.0] | | | 40.0 [10.0] | | | >0.99 | | |  |
| Depression | 35.0 [15.0] | | | 30.0 [7.5] | | | 0.42 | | |  |
| Anger | 25.0 [37.5] | | | 37.5 [35.0] | | | 0.58 | | |  |
| Pain | 20.0 [0] | | | 24.0 [6.0] | | | 0.79 | | |  |
| Fatigue | 37.5 [10.0] | | | 30.0 [20.0] | | | 0.36 | | |  |
| Stigma | 25.0 [2.5] | | | 25.0 [7.5] | | | 0.59 | | |  |
| Cognitive function | 45.0 [2.5] | | | 52.5 [22.5] | | | 0.18 | | |  |
| Total impairment score (QOL-I) | 39.0 [7.92] | | | 33.0 [8.48] | | | 0.63 | | |  |

**Supplementary Table 1: Evolution of impairment scores with paired analysis**

Data are represented as median [IQR].

Bolded values indicate statistical significance at p < 0.05.
