# Supplementary figures and images for: Quality of life over time after new onset refractory status epilepticus
Source: Epilepsia. 2025 Sep 13;67(1):328–40. doi: 10.1111/epi.18635 (PMC12893261; doi:10.1111/epi.18635)

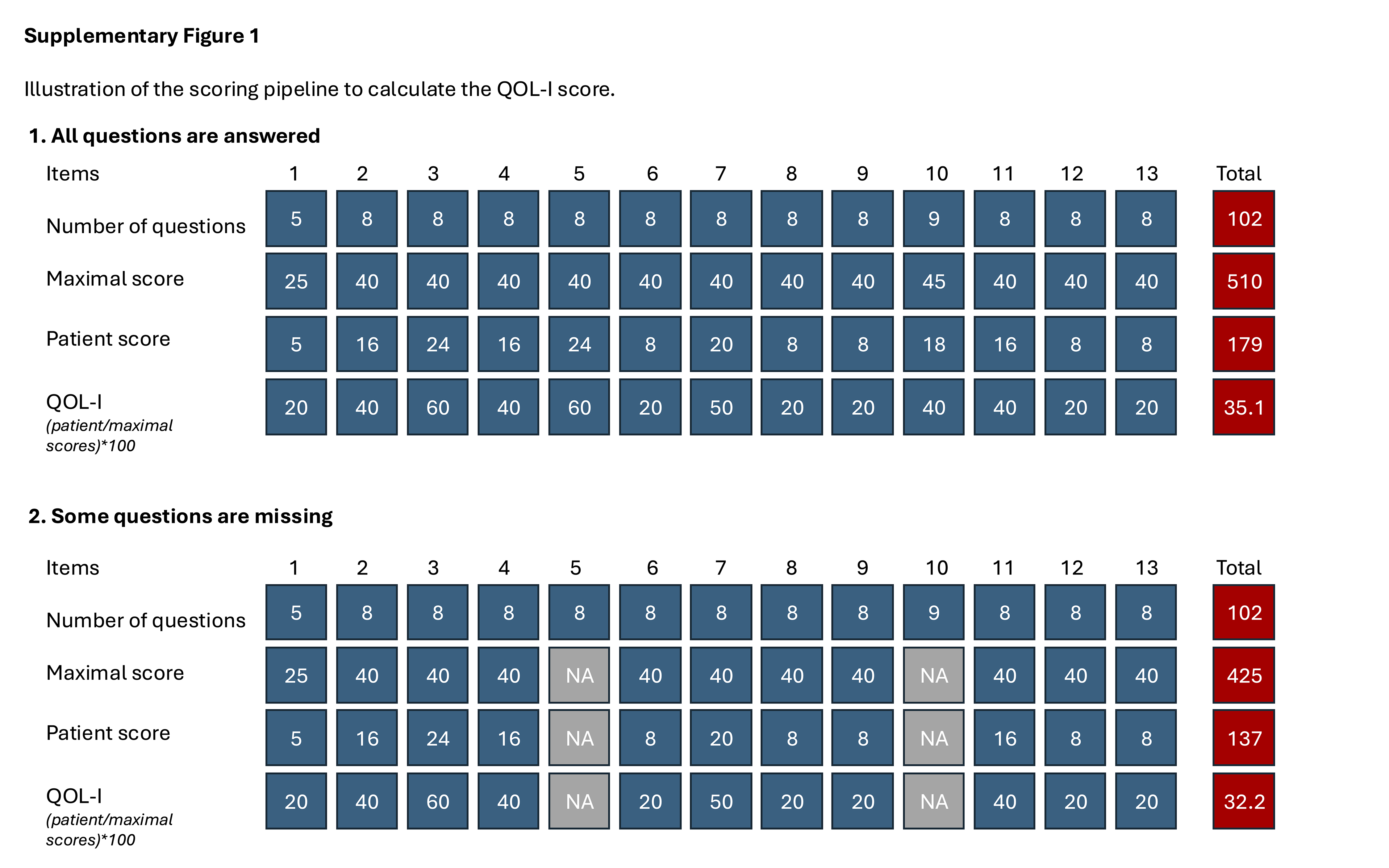

Supplement: Supplementary file 2 — Figure S1. [file EPI-67-328-s003.tif]
