# Supplementary material for: Quality of life over time after new onset refractory status epilepticus
Source: Epilepsia. 2025 Sep 13;67(1):328–40. doi: 10.1111/epi.18635 (PMC12893261; doi:10.1111/epi.18635)
Supplement: Supplementary file 3 — Figure S2. [file EPI-67-328-s002.pptx]

## Slide 1
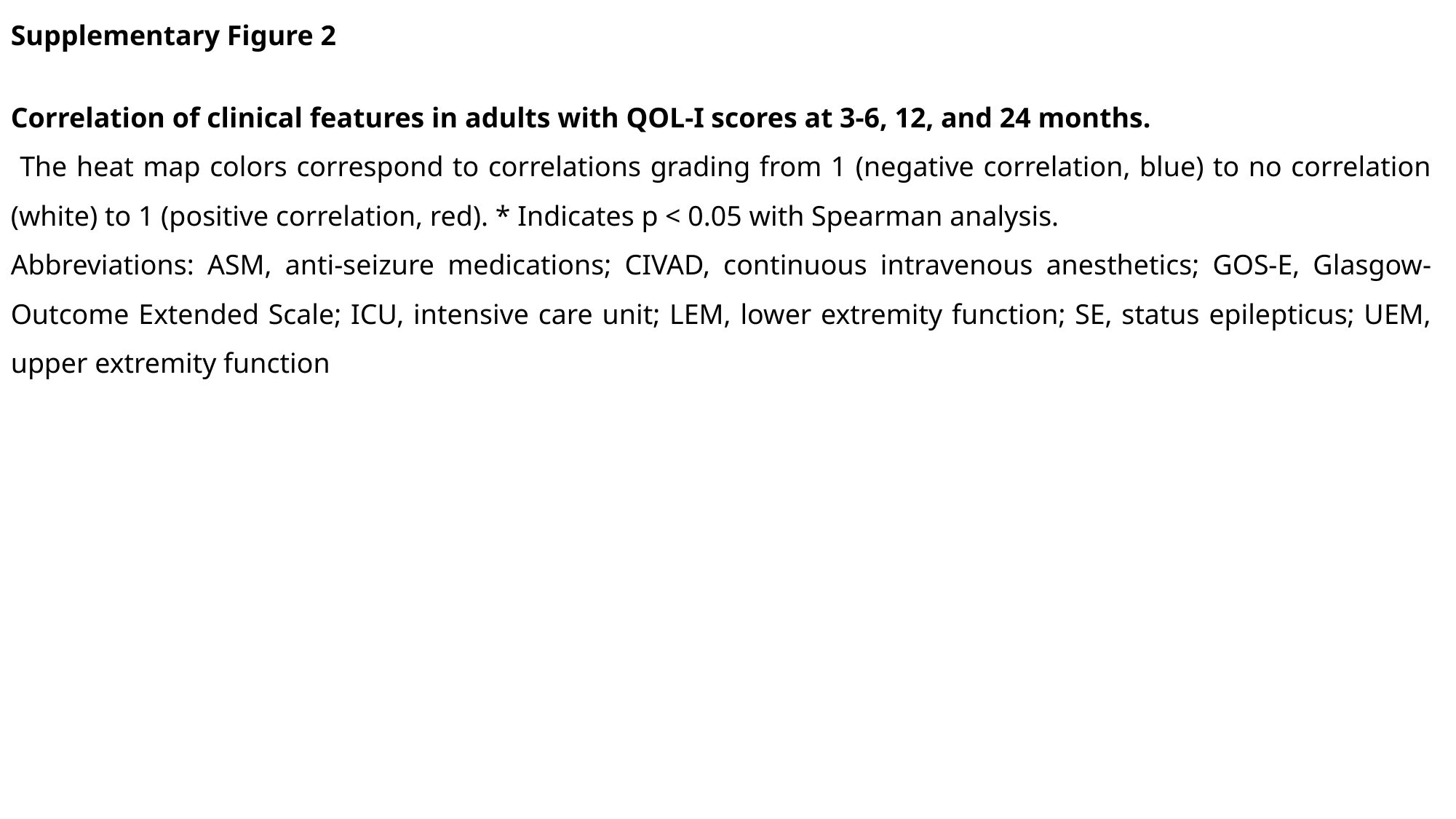

Supplementary Figure 2
Correlation of clinical features in adults with QOL-I scores at 3-6, 12, and 24 months.
 The heat map colors correspond to correlations grading from 1 (negative correlation, blue) to no correlation (white) to 1 (positive correlation, red). * Indicates p < 0.05 with Spearman analysis.
Abbreviations: ASM, anti-seizure medications; CIVAD, continuous intravenous anesthetics; GOS-E, Glasgow-Outcome Extended Scale; ICU, intensive care unit; LEM, lower extremity function; SE, status epilepticus; UEM, upper extremity function

## Slide 2
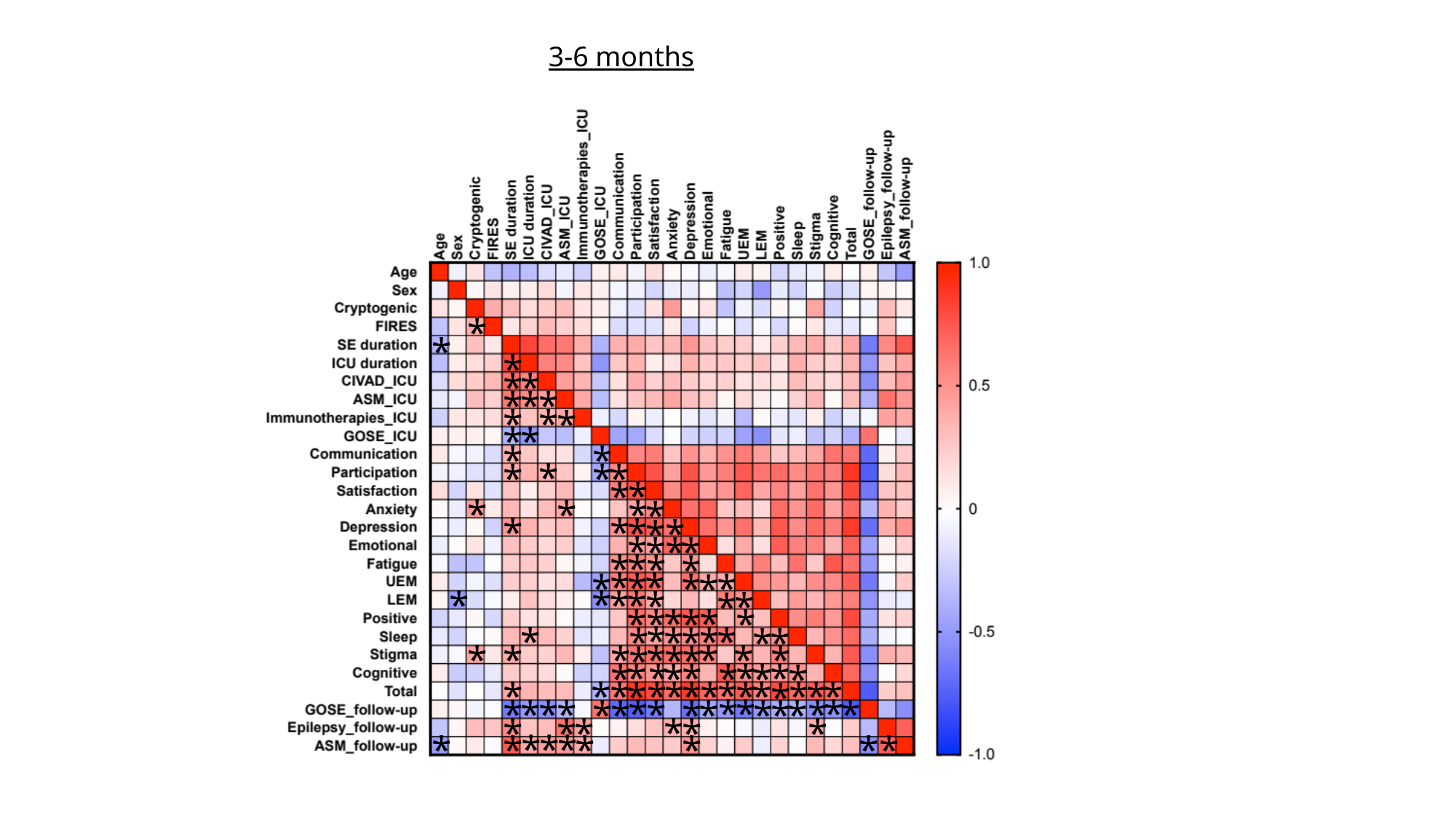

3-6 months
*
*
*
*
*
*
*
*
*
*
*
*
*
*
*
*
*
*
*
*
*
*
*
*
*
*
*
*
*
*
*
*
*
*
*
*
*
*
*
*
*
*
*
*
*
*
*
*
*
*
*
*
*
*
*
*
*
*
*
*
*
*
*
*
*
*
*
*
*
*
*
*
*
*
*
*
*
*
*
*
*
*
*
*
*
*
*
*
*
*
*
*
*
*
*
*
*
*
*
*
*
*
*
*
*
*
*
*
*
*
*
*
*
*
*
*
*
*
*
*
*
*
*
*
*
*
*
*
*
*
*
*
*
*
*

## Slide 3
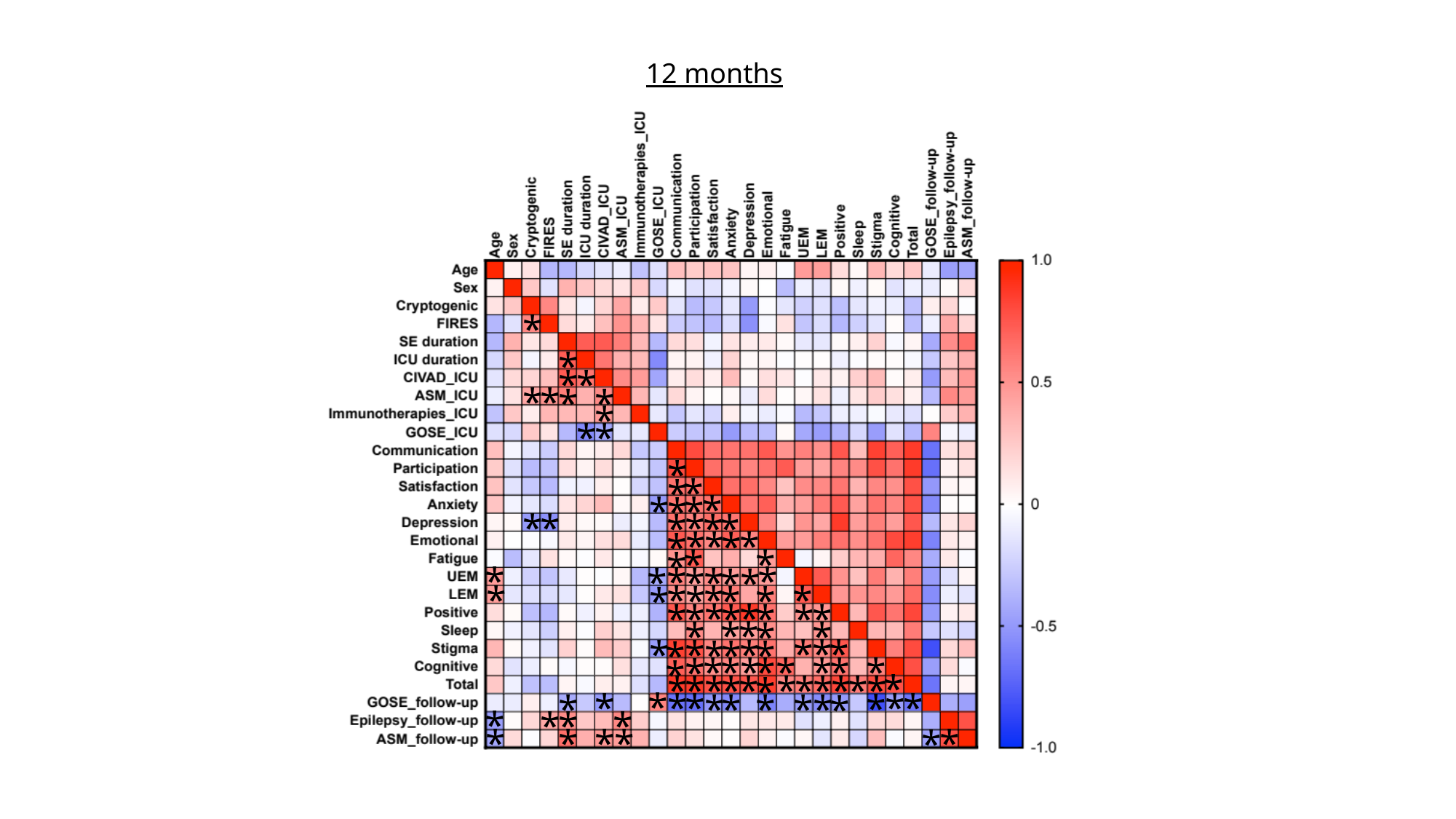

12 months
*
*
*
*
*
*
*
*
*
*
*
*
*
*
*
*
*
*
*
*
*
*
*
*
*
*
*
*
*
*
*
*
*
*
*
*
*
*
*
*
*
*
*
*
*
*
*
*
*
*
*
*
*
*
*
*
*
*
*
*
*
*
*
*
*
*
*
*
*
*
*
*
*
*
*
*
*
*
*
*
*
*
*
*
*
*
*
*
*
*
*
*
*
*
*
*
*
*
*
*
*
*
*
*
*
*
*
*
*
*
*
*
*
*
*
*
*
*

## Slide 4
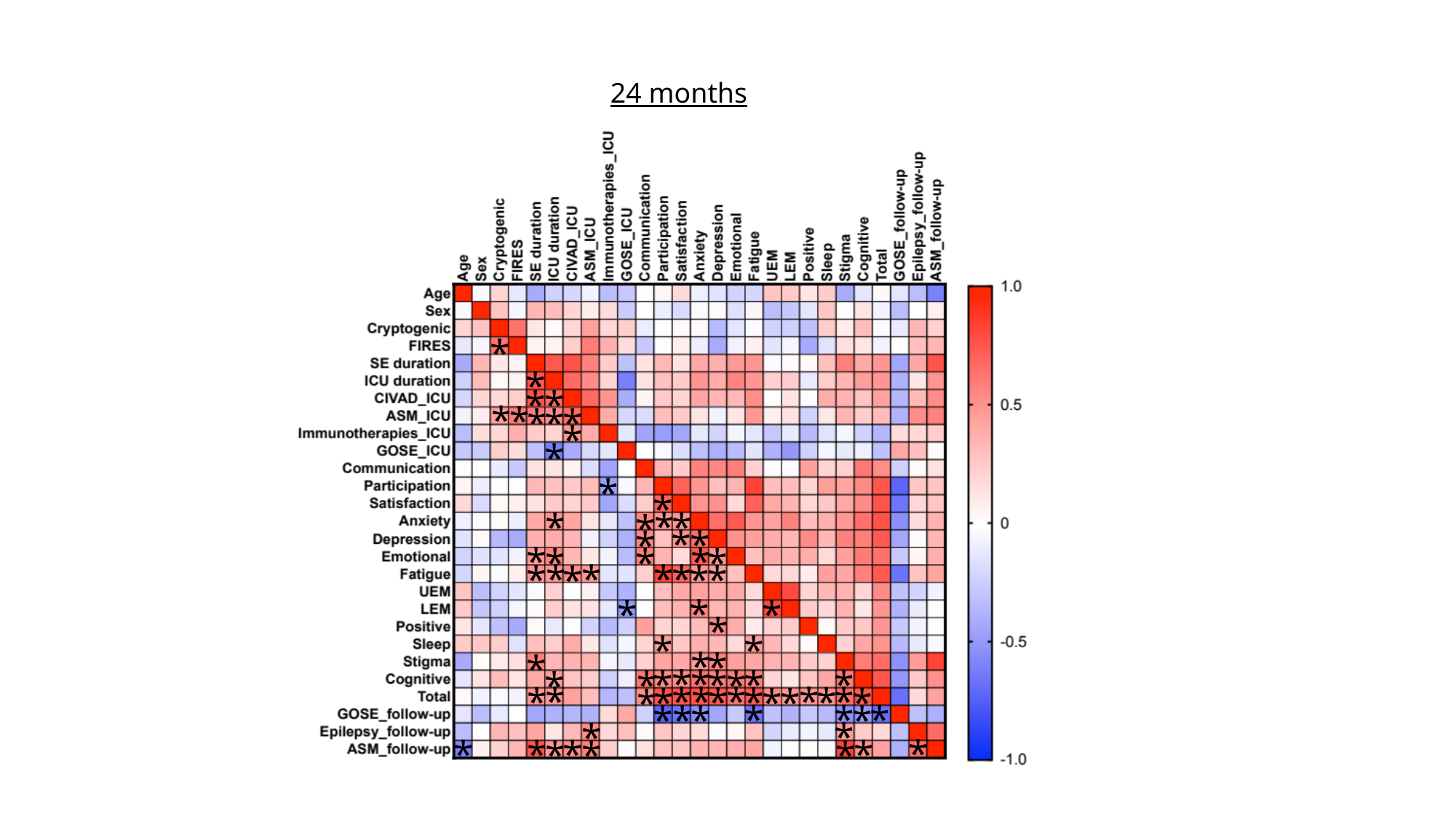

24 months
*
*
*
*
*
*
*
*
*
*
*
*
*
*
*
*
*
*
*
*
*
*
*
*
*
*
*
*
*
*
*
*
*
*
*
*
*
*
*
*
*
*
*
*
*
*
*
*
*
*
*
*
*
*
*
*
*
*
*
*
*
*
*
*
*
*
*
*
*
*
*
*
*
*
*
*
*
*
*
*
*
*
*
