# Supplementary material for: Quality of life over time after new onset refractory status epilepticus
Source: Epilepsia. 2025 Sep 13;67(1):328–40. doi: 10.1111/epi.18635 (PMC12893261; doi:10.1111/epi.18635)
Supplement: Supplementary file 4 — Data S1. [file EPI-67-328-s001.pdf]

PATIENT ID: ___________________

DATE: _________________________

Communication – Short Form

Please respond to each question or statement by marking one box per row.

	**How much DIFFICULTY do you currently have…**	**None**	**A little**	**Somewhat**	**A lot**	**Cannot do**
NQCOG01	writing notes to yourself, such as appointments or 'to do' lists?............................	☐ 5	☐ 4	☐ 3	☐ 2	☐ 1
NQCOG04	understanding family and friends on the phone?..	☐ 5	☐ 4	☐ 3	☐ 2	☐ 1
NQCOG08	carrying on a conversation with a small group of familiar people (e.g., family or a few friends)?..	☐ 5	☐ 4	☐ 3	☐ 2	☐ 1
NQCOG10	organizing what you want to say?...................	☐ 5	☐ 4	☐ 3	☐ 2	☐ 1
NQCOG11	speaking clearly enough to use the telephone?..	☐ 5	☐ 4	☐ 3	☐ 2	☐ 1

©2008-2013 David Cella and the PROMIS Health Organization on behalf of the National Institute for Neurological Disorders and Stroke (NINDS). Used with permission.

Ability to Participate in Social Roles and Activities – Short Form

Please respond to each question or statement by marking one box per row.

	**In the past 7 days…**	**Never**	**Rarely**	**Sometimes**	**Often**	**Always**
NQPRF01	I can keep up with my family responsibilities……………………………	☐ 1	☐ 2	☐ 3	☐ 4	☐ 5
NQPRF03	I am able to do all of my regular family activities………………………………….	☐ 1	☐ 2	☐ 3	☐ 4	☐ 5
NQPRF08	I am able to socialize with my friends…...	☐ 1	☐ 2	☐ 3	☐ 4	☐ 5
NQPRF09	I am able to do all of my regular activities with friends……………………………….	☐ 1	☐ 2	☐ 3	☐ 4	☐ 5
NQPRF17	I can keep up with my social commitments……………………………..	☐ 1	☐ 2	☐ 3	☐ 4	☐ 5
NQPRF26	I am able to participate in leisure activities………………………………….	☐ 1	☐ 2	☐ 3	☐ 4	☐ 5
NQPRF32	I am able to perform my daily routines…..	☐ 1	☐ 2	☐ 3	☐ 4	☐ 5
NQPRF34	I can keep up with my work responsibilities (include work at home)….	☐ 1	☐ 2	☐ 3	☐ 4	☐ 5

©2008-2013 David Cella and the PROMIS Health Organization on behalf of the National Institute for Neurological Disorders and Stroke (NINDS). Used with permission.

Anxiety – Short Form

Please respond to each question or statement by marking one box per row.

	In the past 7 days…	Never	Rarely	Sometimes	Often	Always
NQANX26	I felt uneasy………………………………	☐ 1	☐ 2	☐ 3	☐ 4	☐ 5
NQANX22	I felt nervous…………………………...	☐ 1	☐ 2	☐ 3	☐ 4	☐ 5
NQANX23	Many situations made me worry…………	☐ 1	☐ 2	☐ 3	☐ 4	☐ 5
NQANX20	My worries overwhelmed me…………….	☐ 1	☐ 2	☐ 3	☐ 4	☐ 5
NQANX27	I felt tense……………………………...	☐ 1	☐ 2	☐ 3	☐ 4	☐ 5
NQANX28	I had difficulty calming down….………...	☐ 1	☐ 2	☐ 3	☐ 4	☐ 5
NQANX09	I had sudden feelings of panic……………	☐ 1	☐ 2	☐ 3	☐ 4	☐ 5
NQANX07	I felt nervous when my normal routine was disturbed…………………….…….	☐ 1	☐ 2	☐ 3	☐ 4	☐ 5

©2008-2013 David Cella and the PROMIS Health Organization on behalf of the National Institute for Neurological Disorders and Stroke (NINDS). Used with permission.

Depression – Short Form

Please respond to each question or statement by marking one box per row.

	In the past 7 days…	Never	Rarely	Sometimes	Often	Always
NQDEP13	I felt depressed…………………………...	☐ 1	☐ 2	☐ 3	☐ 4	☐ 5
NQDEP23	I felt hopeless…………………………….	☐ 1	☐ 2	☐ 3	☐ 4	☐ 5
NQDEP07	I felt that nothing could cheer me up……..	☐ 1	☐ 2	☐ 3	☐ 4	☐ 5
NQDEP27	I felt that my life was empty……………...	☐ 1	☐ 2	☐ 3	☐ 4	☐ 5
NQDEP02	I felt worthless……………………………	☐ 1	☐ 2	☐ 3	☐ 4	☐ 5
NQDEP19	I felt unhappy…………………………….	☐ 1	☐ 2	☐ 3	☐ 4	☐ 5
NQDEP21	I felt I had no reason for living…………...	☐ 1	☐ 2	☐ 3	☐ 4	☐ 5
NQDEP24	I felt that nothing was interesting………...	☐ 1	☐ 2	☐ 3	☐ 4	☐ 5

©2008-2013 David Cella and the PROMIS Health Organization on behalf of the National Institute for Neurological Disorders and Stroke (NINDS). Used with permission.

Emotional and Behavioral Dyscontrol – Short Form

Please respond to each question or statement by marking one box per row.

	**In the past 7 days…**	**Never**	**Rarely**	**Sometimes**	**Often**	**Always**
NQPER02	I had trouble controlling my temper……..	☐ 1	☐ 2	☐ 3	☐ 4	☐ 5
NQPER05	It was hard to control my behavior……….	☐ 1	☐ 2	☐ 3	☐ 4	☐ 5
NQPER06	I said or did things without thinking……..	☐ 1	☐ 2	☐ 3	☐ 4	☐ 5
NQPER07	I got impatient with other people………..	☐ 1	☐ 2	☐ 3	☐ 4	☐ 5
NQPER11	I was irritable around other people……….	☐ 1	☐ 2	☐ 3	☐ 4	☐ 5
NQPER12	I was bothered by little things……………	☐ 1	☐ 2	☐ 3	☐ 4	☐ 5
NQPER17	I became easily upset……………………..	☐ 1	☐ 2	☐ 3	☐ 4	☐ 5
NQPER19	I was in conflict with others……………..	☐ 1	☐ 2	☐ 3	☐ 4	☐ 5

©2008-2013 David Cella and the PROMIS Health Organization on behalf of the National Institute for Neurological Disorders and Stroke (NINDS). Used with permission.

Fatigue – Short Form

Please respond to each question or statement by marking one box per row.

	**In the past 7 days…**	**Never**	**Rarely**	**Sometimes**	**Often**	**Always**
NQFTG13	I felt exhausted…………………………...	☐ 1	☐ 2	☐ 3	☐ 4	☐ 5
NQFTG11	I felt that I had no energy………………...	☐ 1	☐ 2	☐ 3	☐ 4	☐ 5
NQFTG15	I felt fatigued……………………………..	☐ 1	☐ 2	☐ 3	☐ 4	☐ 5
NQFTG06	I was too tired to do my household chores.	☐ 1	☐ 2	☐ 3	☐ 4	☐ 5
NQFTG07	I was too tired to leave the house………...	☐ 1	☐ 2	☐ 3	☐ 4	☐ 5
NQFTG10	I was frustrated by being too tired to do the things I wanted to do…………………	☐ 1	☐ 2	☐ 3	☐ 4	☐ 5
NQFTG14	I felt tired…………………………………	☐ 1	☐ 2	☐ 3	☐ 4	☐ 5
NQFTG02	I had to limit my social activity because I was tired…………………………………	☐ 1	☐ 2	☐ 3	☐ 4	☐ 5

©2008-2013 David Cella and the PROMIS Health Organization on behalf of the National Institute for Neurological Disorders and Stroke (NINDS). Used with permission.

Lower Extremity Function (Mobility) – Short Form

Please respond to each question or statement by marking one box per row.

		Without any difficulty	With a little difficulty	With some difficulty	With much difficulty	Unable to do
NQMOB37	Are you able to get on and off the toilet?...	☐ 5	☐ 4	☐ 3	☐ 2	☐ 1
NQMOB30	Are you able to step up and down curbs?...	☐ 5	☐ 4	☐ 3	☐ 2	☐ 1
NQMOB26	Are you able to get in and out of a car?.....	☐ 5	☐ 4	☐ 3	☐ 2	☐ 1
NQMOB32	Are you able to get out of bed into a chair?...	☐ 5	☐ 4	☐ 3	☐ 2	☐ 1
NQMOB25	Are you able to push open a heavy door?..	☐ 5	☐ 4	☐ 3	☐ 2	☐ 1
NQMOB33	Are you able to run errands and shop?.......	☐ 5	☐ 4	☐ 3	☐ 2	☐ 1
NQMOB31	Are you able to get up off the floor from lying on your back without help?..............	☐ 5	☐ 4	☐ 3	☐ 2	☐ 1
NQMOB28	Are you able to go for a walk of at least 15 minutes?...	☐ 5	☐ 4	☐ 3	☐ 2	☐ 1

©2008-2013 David Cella and the PROMIS Health Organization on behalf of the National Institute for Neurological Disorders and Stroke (NINDS). Used with permission.

Positive Affect and Well-Being - Short Form

Please respond to each question or statement by marking one box per row.

	Lately…	Never	Rarely	Sometimes	Often	Always
NQPPF14	I had a sense of well-being………………	☐ 1	☐ 2	☐ 3	☐ 4	☐ 5
NQPPF12	I felt hopeful……………………………..	☐ 1	☐ 2	☐ 3	☐ 4	☐ 5
NQPPF15	My life was satisfying……………………	☐ 1	☐ 2	☐ 3	☐ 4	☐ 5
NQPPF20	My life had purpose………………………	☐ 1	☐ 2	☐ 3	☐ 4	☐ 5
NQPPF17	My life had meaning……………………..	☐ 1	☐ 2	☐ 3	☐ 4	☐ 5
NQPPF22	I felt cheerful……………………………..	☐ 1	☐ 2	☐ 3	☐ 4	☐ 5
NQPPF19	My life was worth living…………………	☐ 1	☐ 2	☐ 3	☐ 4	☐ 5
NQPPF16	I had a sense of balance in my life………	☐ 1	☐ 2	☐ 3	☐ 4	☐ 5
NQPPF07	Many areas of my life were interesting to me……………………………………...	☐ 1	☐ 2	☐ 3	☐ 4	☐ 5

©2008-2013 David Cella and the PROMIS Health Organization on behalf of the National Institute for Neurological Disorders and Stroke (NINDS). Used with permission.

Sleep Disturbance – Short Form

Please respond to each question or statement by marking one box per row.

	**In the past 7 days…**	**Never**	**Rarely**	**Sometimes**	**Often**	**Always**
NQSLP02	I had to force myself to get up in the morning…………………………………..	☐ 1	☐ 2	☐ 3	☐ 4	☐ 5
NQSLP03	I had trouble stopping my thoughts at bedtime…………………………………...	☐ 1	☐ 2	☐ 3	☐ 4	☐ 5
NQSLP04	I was sleepy during the daytime…………	☐ 1	☐ 2	☐ 3	☐ 4	☐ 5
NQSLP05	I had trouble sleeping because of bad dreams……………………………………	☐ 1	☐ 2	☐ 3	☐ 4	☐ 5
NQSLP07	I had trouble falling asleep………………	☐ 1	☐ 2	☐ 3	☐ 4	☐ 5
NQSLP12	Pain woke me up…………………………	☐ 1	☐ 2	☐ 3	☐ 4	☐ 5
NQSLP13	I avoided or cancelled activities with my friends because I was tired from having a bad night's sleep………………………….	☐ 1	☐ 2	☐ 3	☐ 4	☐ 5
NQSLP18	I felt physically tense during the middle of the night or early morning hours………..	☐ 1	☐ 2	☐ 3	☐ 4	☐ 5

©2008-2013 David Cella and the PROMIS Health Organization on behalf of the National Institute for Neurological Disorders and Stroke (NINDS). Used with permission.

Upper Extremity Function (Fine Motor, ADL) – Short Form

Please respond to each question or statement by marking one box per row.

		Without any difficulty	With a little difficulty	With some difficulty	With much difficulty	Unable to do
NQUEX29	Are you able to turn a key in a lock?..........	☐ 5	☐ 4	☐ 3	☐ 2	☐ 1
NQUEX20	Are you able to brush your teeth?.............	☐ 5	☐ 4	☐ 3	☐ 2	☐ 1
NQUEX44	Are you able to make a phone call using a touch tone key-pad?..................................	☐ 5	☐ 4	☐ 3	☐ 2	☐ 1
NQUEX36	Are you able to pick up coins from a table top?..	☐ 5	☐ 4	☐ 3	☐ 2	☐ 1
NQUEX30	Are you able to write with a pen or pencil?..	☐ 5	☐ 4	☐ 3	☐ 2	☐ 1
NQUEX28	Are you able to open and close a zipper?...	☐ 5	☐ 4	☐ 3	☐ 2	☐ 1
NQUEX33	Are you able to wash and dry your body?..	☐ 5	☐ 4	☐ 3	☐ 2	☐ 1
NQUEX37	Are you able to shampoo your hair?..........	☐ 5	☐ 4	☐ 3	☐ 2	☐ 1

©2008-2013 David Cella and the PROMIS Health Organization on behalf of the National Institute for Neurological Disorders and Stroke (NINDS). Used with permission.

Stigma-Short Form

Please respond to each question or statement by marking one box per row.

	**Lately…**	**Never**	**Rarely**	**Sometimes**	**Often**	**Always**
NQSTG02	Because of my illness, some people avoided me………………………………	☐ 1	☐ 2	☐ 3	☐ 4	☐ 5
NQSTG04	Because of my illness, I felt left out of things……………………………………	☐ 1	☐ 2	☐ 3	☐ 4	☐ 5
NQSTG 08	Because of my illness, people avoided looking at me……………………………	☐ 1	☐ 2	☐ 3	☐ 4	☐ 5
NQSTG 16	I felt embarrassed about my illness……..	☐ 1	☐ 2	☐ 3	☐ 4	☐ 5
NQSTG 01	Because of my illness, some people seemed uncomfortable with me………..	☐ 1	☐ 2	☐ 3	☐ 4	☐ 5
NQSTG 17	I felt embarrassed because of my physical limitations…..……………………………	☐ 1	☐ 2	☐ 3	☐ 4	☐ 5
NQSTG05	Because of my illness, people were unkind to me …………………………..	☐ 1	☐ 2	☐ 3	☐ 4	☐ 5
NQSTG21	Some people acted as though it was my fault I have this illness..…………………	☐ 1	☐ 2	☐ 3	☐ 4	☐ 5

©2008-2013 David Cella and the PROMIS Health Organization on behalf of the National Institute for Neurological Disorders and Stroke (NINDS). Used with permission.

Satisfaction with Social Roles and Activities – Short Form

Please respond to each question or statement by marking one box per row.

	In the past 7 days…	Not at all	A little bit	Somewhat	Quite a bit	Very much
NQSAT 03	I am bothered by my limitations in regular family activities..	☐ 5	☐ 4	☐ 3	☐ 2	☐ 1
NQSAT 23	I am disappointed in my ability to socialize with my family………………...	☐ 5	☐ 4	☐ 3	☐ 2	☐ 1
NQSAT14	I am bothered by limitations in my regular activities with friends.……………………	☐ 5	☐ 4	☐ 3	☐ 2	☐ 1
NQSAT11	I am disappointed in my ability to meet the needs of my friends	☐ 5	☐ 4	☐ 3	☐ 2	☐ 1

	In the past 7 days…	Not at all	A little bit	Somewhat	Quite a bit	Very much
NQSAT33	I am satisfied with my ability to do things for fun outside my home………………....	☐ 1	☐ 2	☐ 3	☐ 4	☐ 5
NQSAT32	I am satisfied with the amount of time I spend doing leisure activities…………….	☐ 1	☐ 2	☐ 3	☐ 4	☐ 5
NQSAT47	I am satisfied with how much of my work I can do (include work at home).…………	☐ 1	☐ 2	☐ 3	☐ 4	☐ 5
NQSAT 46	I am satisfied with my ability to do household chores or tasks…………….........	☐ 1	☐ 2	☐ 3	☐ 4	☐ 5

©2008-2013 David Cella and the PROMIS Health Organization on behalf of the National Institute for Neurological Disorders and Stroke (NINDS). Used with permission.

Cognition Function– Short Form

Please respond to each question or statement by marking one box per row.

	In the past 7 days…	Never	Rarely (once)	Sometimes (2-3 times)	Often (once a day)	Very often (several times a day)
NQCOG64	I had to read something several times to understand it………………………………...	☐ 5	☐ 4	☐ 3	☐ 2	☐ 1
NQCOG75	My thinking was slow……………………	☐ 5	☐ 4	☐ 3	☐ 2	☐ 1
NQCOG77	I had to work really hard to pay attention or I would make a mistake……………….	☐ 5	☐ 4	☐ 3	☐ 2	☐ 1
NQCOG80	I had trouble concentrating……………….	☐ 5	☐ 4	☐ 3	☐ 2	☐ 1

	How much DIFFICULTY do you currently have…	None	A little	Somewhat	A lot	Cannot do
NQCOG22	reading and following complex instructions (e.g., directions for a new medication)?...	☐ 5	☐ 4	☐ 3	☐ 2	☐ 1
NQCOG24	planning for and keeping appointments that are not part of your weekly routine, (e.g., a therapy or doctor appointment, or a social gathering with friends and family)?..	☐ 5	☐ 4	☐ 3	☐ 2	☐ 1
NQCOG25	managing your time to do most of your daily activities?...	☐ 5	☐ 4	☐ 3	☐ 2	☐ 1
NQCOG40	learning new tasks or instructions?...........	☐ 5	☐ 4	☐ 3	☐ 2	☐ 1

©2008-2013 David Cella and the PROMIS Health Organization on behalf of the National Institute for Neurological Disorders and Stroke (NINDS). Used with permission.

PATIENT ID: ____________________

DATE: __________________________

Neuro-QOL Pediatric Short Forms

Social Relations - Interaction with Peers

Please respond to each question or statement by marking one box per row.

	**In the past 7 days…**	**Never**	**Almost never**	**Sometimes**	**Often**	**Almost always**
NQSCLped11	I felt close to my friends.	☐ 1	☐ 2	☐ 3	☐ 4	☐ 5
NQSCLped12	I was able to count on my friends.……...	☐ 1	☐ 2	☐ 3	☐ 4	☐ 5
NQSCLped20	I felt comfortable with others my age…...	☐ 1	☐ 2	☐ 3	☐ 4	☐ 5
NQSCLped28	I was happy with the friends I had.	☐ 1	☐ 2	☐ 3	☐ 4	☐ 5
NQSCLped30	I felt comfortable talking with my friends…………………………………..	☐ 1	☐ 2	☐ 3	☐ 4	☐ 5
NQSCLped32	I spent time with my friends.…………...	☐ 1	☐ 2	☐ 3	☐ 4	☐ 5
NQSCLped36	My friends and I helped each other out………………………………………	☐ 1	☐ 2	☐ 3	☐ 4	☐ 5
NQSCLped38	I had fun with my friends.………………	☐ 1	☐ 2	☐ 3	☐ 4	☐ 5

©2008-2013 David Cella and the PROMIS Health Organization on behalf of the National Institute for Neurological Disorders and Stroke (NINDS). Used with permission.

Pediatric Anxiety – Short Form

Please respond to each question or statement by marking one box per row.

	In the past 7 days…	Never	Almost never	Sometimes	Often	Almost always
NQEMNped22	I felt afraid to go out alone.	☐ 1	☐ 2	☐ 3	☐ 4	☐ 5
NQEMNped23	Being worried made it hard for me to be with my friends.………………………….	☐ 1	☐ 2	☐ 3	☐ 4	☐ 5
NQEMNped24	It was hard to do schoolwork because I was nervous or worried………………….	☐ 1	☐ 2	☐ 3	☐ 4	☐ 5
NQEMNped26	I felt afraid………………………………	☐ 1	☐ 2	☐ 3	☐ 4	☐ 5
NQEMNped28	I worried when I was at home.………......	☐ 1	☐ 2	☐ 3	☐ 4	☐ 5
NQEMNped29	I felt worried.…………………………….	☐ 1	☐ 2	☐ 3	☐ 4	☐ 5

	In the past 7 days…	Not at all	A little bit	Somewhat	Quite a bit	Very much
NQEMNped43	I worry that my health might get worse.....	☐ 1	☐ 2	☐ 3	☐ 4	☐ 5
NQEMNped46	I worry about doing well in school..…......	☐ 1	☐ 2	☐ 3	☐ 4	☐ 5

©2008-2013 David Cella and the PROMIS Health Organization on behalf of the National Institute for Neurological Disorders and Stroke (NINDS). Used with permission.

Pediatric Depression – Short Form

Please respond to each question or statement by marking one box per row.

	In the past 7 days…	Never	Almost never	Sometimes	Often	Almost always
NQEMNped01	I felt too sad to do things with friends……	☐ 1	☐ 2	☐ 3	☐ 4	☐ 5
NQEMNped04	I felt sad…………………………………	☐ 1	☐ 2	☐ 3	☐ 4	☐ 5
NQEMNped09	I felt lonely.	☐ 1	☐ 2	☐ 3	☐ 4	☐ 5
NQEMNped31	I was less interested in doing things I usually enjoy……………………………..	☐ 1	☐ 2	☐ 3	☐ 4	☐ 5
NQEMNped34	It was hard for me to care about anything………………………………….	☐ 1	☐ 2	☐ 3	☐ 4	☐ 5
NQEMNped36	It was hard for me to have fun…………..	☐ 1	☐ 2	☐ 3	☐ 4	☐ 5
NQEMNped40	I felt like I couldn't do anything right……	☐ 1	☐ 2	☐ 3	☐ 4	☐ 5
NQEMNped41	I felt everything in my life went wrong.…	☐ 1	☐ 2	☐ 3	☐ 4	☐ 5

©2008-2013 David Cella and the PROMIS Health Organization on behalf of the National Institute for Neurological Disorders and Stroke (NINDS). Used with permission.

Pediatric Anger– Short Form

Please respond to each question or statement by marking one box per row.

	**In the past 7 days…**	**Never**	**Almost never**	**Sometimes**	**Often**	**Almost always**
NQEMNped12	Being angry made it hard for me to be with my friends.	☐ 1	☐ 2	☐ 3	☐ 4	☐ 5
NQEMNped13	It was hard to do schoolwork because I was angry………………………………	☐ 1	☐ 2	☐ 3	☐ 4	☐ 5
NQEMNped14	I felt angry	☐ 1	☐ 2	☐ 3	☐ 4	☐ 5
NQEMNped15	I was so mad that I felt like throwing something……..	☐ 1	☐ 2	☐ 3	☐ 4	☐ 5
NQEMNped16	I was so mad that I felt like hitting something………………………………	☐ 1	☐ 2	☐ 3	☐ 4	☐ 5
NQEMNped17	I was so mad that I felt like yelling at someone………………………………..	☐ 1	☐ 2	☐ 3	☐ 4	☐ 5
NQEMNped18	I was so mad that I felt like breaking things………………………………...	☐ 1	☐ 2	☐ 3	☐ 4	☐ 5
NQEMNped19	I was so mad that I acted grouchy towards other people…………………...	☐ 1	☐ 2	☐ 3	☐ 4	☐ 5

©2008-2013 David Cella and the PROMIS Health Organization on behalf of the National Institute for Neurological Disorders and Stroke (NINDS). Used with permission.

Pediatric Pain– Short Form

Please respond to each question or statement by marking one box per row.

	In the past 7 days…	Never	Almost never	Sometimes	Often	Almost always
NQPAIped01	I had a lot of pain…………………………	☐ 1	☐ 2	☐ 3	☐ 4	☐ 5
NQPAIped02	My pain was so bad that I needed to take medicine for it……………………………	☐ 1	☐ 2	☐ 3	☐ 4	☐ 5
NQPAIped03	I missed school when I had pain………..	☐ 1	☐ 2	☐ 3	☐ 4	☐ 5
NQPAIped04	I had so much pain that I had to stop what I was doing……………………………….	☐ 1	☐ 2	☐ 3	☐ 4	☐ 5
NQPAIped05	I hurt all over my body…………………...	☐ 1	☐ 2	☐ 3	☐ 4	☐ 5
NQPAIped06	I had pain…………………………………	☐ 1	☐ 2	☐ 3	☐ 4	☐ 5
NQPAIped08	I had trouble sleeping when I had pain…..	☐ 1	☐ 2	☐ 3	☐ 4	☐ 5
NQPAIped09	I had trouble watching TV when I had pain………………………………………	☐ 1	☐ 2	☐ 3	☐ 4	☐ 5
NQPAIped10	It was hard for me to play or hang out with my friends when I had pain………..	☐ 1	☐ 2	☐ 3	☐ 4	☐ 5

	In the past 7 days…	Few seconds	Few minutes	Few hours	Few days (less than a week)	More than a week
NQPAIped07	When you had pain, how long did it last?..	☐ 1	☐ 2	☐ 3	☐ 4	☐ 5

©2008-2013 David Cella and the PROMIS Health Organization on behalf of the National Institute for Neurological Disorders and Stroke (NINDS). Used with permission.

Pediatric Fatigue –Short Form

Please respond to each question or statement by marking one box per row.

	**In the past 7 days…**	**None of the time**	**A little bit of time**	**Some of the time**	**Most of the time**	**All of the time**
NQFTGped01	I felt tired………………………………....	☐ 1	☐ 2	☐ 3	☐ 4	☐ 5
NQFTGped04	I had trouble starting things because I was too tired…………………………………..	☐ 1	☐ 2	☐ 3	☐ 4	☐ 5
NQFTGped05	I had trouble finishing things because I was too tired……………………………..	☐ 1	☐ 2	☐ 3	☐ 4	☐ 5
NQFTGped06	I needed to sleep during the day………..	☐ 1	☐ 2	☐ 3	☐ 4	☐ 5
NQFTGped08	Being tired made it hard to play or go out with my friends as much as I would like...	☐ 1	☐ 2	☐ 3	☐ 4	☐ 5
NQFTGped11r1	I was too tired to eat……………………..	☐ 1	☐ 2	☐ 3	☐ 4	☐ 5
NQFTGped12	Being tired makes me sad………………..	☐ 1	☐ 2	☐ 3	☐ 4	☐ 5
NQFTGped13	Being tired makes me mad………………	☐ 1	☐ 2	☐ 3	☐ 4	☐ 5

©2008-2013 David Cella and the PROMIS Health Organization on behalf of the National Institute for Neurological Disorders and Stroke (NINDS). Used with permission.

Pediatric Stigma – Short Form

Please respond to each question or statement by marking one box per row.

	**Lately…**	**Never**	**Rarely**	**Sometimes**	**Often**	**Always**
NQSTGped03	Because of my illness, others my age avoided me...	☐ 1	☐ 2	☐ 3	☐ 4	☐ 5
NQSTGped04	Because of my illness, I felt left out of things.……………………………………	☐ 1	☐ 2	☐ 3	☐ 4	☐ 5
NQSTGped06	Because of my illness, others my age made fun of me…………………………..	☐ 1	☐ 2	☐ 3	☐ 4	☐ 5
NQSTGped07	Because of my illness, I felt embarrassed when I was in front of others my age……	☐ 1	☐ 2	☐ 3	☐ 4	☐ 5
NQSTGped10	Because of my illness, I was treated unfairly by others my age.……………….	☐ 1	☐ 2	☐ 3	☐ 4	☐ 5
NQSTGped13	Because of my illness, others my age tended to ignore my good points………...	☐ 1	☐ 2	☐ 3	☐ 4	☐ 5
NQSTGped17	Because of my illness, I felt different from others my age.……………………...	☐ 1	☐ 2	☐ 3	☐ 4	☐ 5
NQSTGped19	I avoided making new friends to avoid talking about my illness.…………………	☐ 1	☐ 2	☐ 3	☐ 4	☐ 5

©2008-2013 David Cella and the PROMIS Health Organization on behalf of the National Institute for Neurological Disorders and Stroke (NINDS). Used with permission.

Pediatric Cognitive Function – Short Form

Please respond to each question or statement by marking one box per row.

		Not at all	A little bit	Somewhat	Quite a bit	Very much
NQCOGped03	I forget schoolwork that I need to do…... ..	☐ 5	☐ 4	☐ 3	☐ 2	☐ 1
NQCOGped05	I sometimes forget what I was going to say……………………………………..	☐ 5	☐ 4	☐ 3	☐ 2	☐ 1
NQCOGped08	I react slower than most people my age when I play games.	☐ 5	☐ 4	☐ 3	☐ 2	☐ 1
NQCOGped15	I forget things easily.................................	☐ 5	☐ 4	☐ 3	☐ 2	☐ 1
NQCOGped17	I have trouble remembering to do things (e.g., school projects)…………………..	☐ 5	☐ 4	☐ 3	☐ 2	☐ 1
NQCOGped18	It is hard for me to concentrate in school…………………………………..	☐ 5	☐ 4	☐ 3	☐ 2	☐ 1
NQCOGped19	I have trouble paying attention to the teacher………………………………….	☐ 5	☐ 4	☐ 3	☐ 2	☐ 1
NQCOGped20	I have to work really hard to pay attention or I will make a mistake.……..	☐ 5	☐ 4	☐ 3	☐ 2	☐ 1

©2008-2013 David Cella and the PROMIS Health Organization on behalf of the National Institute for Neurological Disorders and Stroke (NINDS). Used with permission.
